# Strand break-induced replication fork collapse leads to C-circles, C-overhangs and telomeric recombination

**DOI:** 10.1371/journal.pgen.1007925

**Published:** 2019-02-04

**Authors:** Tianpeng Zhang, Zepeng Zhang, Gong Shengzhao, Xiaocui Li, Haiying Liu, Yong Zhao

**Affiliations:** 1 MOE Key Laboratory of Gene Function and Regulation, State Key Laboratory of Biocontrol, School of Life Sciences, Sun Yat-sen University, Guangzhou, P. R. China; 2 School of Chemical Engineering and Technology, Guangdong Engineering Technical Research Center for Green Household Chemicals, Guangdong Industry Technical College, Guangzhou, P.R.China; 3 State Key Laboratory of Oncology in South China, Collaborative Innovation Center for Cancer Medicine, Sun Yat-sen University Cancer Center, Guangzhou, P. R. China; Chinese Academy of Sciences, CHINA

## Abstract

Telomerase-independent ALT (alternative lengthening of telomeres) cells are characterized by high frequency of telomeric homologous recombination (HR), C-rich extrachromosomal circles (C-circles) and C-rich terminal 5' overhangs (C-overhangs). However, underlying mechanism is poorly understood. Here, we show that both C-circle and C-overhang form when replication fork collapse is induced by strand break at telomeres. We find that endogenous DNA break predominantly occur on C-rich strand of telomeres in ALT cells, resulting in high frequency of replication fork collapse. While collapsed forks could be rescued by replication fork regression leading to telomeric homologous recombination, those unresolved are converted to C-circles and C-overhang at lagging and leading synthesized strand, respectively. Meanwhile, multiple hallmarks of ALT are provoked, suggesting that strand break-induced replication stress underlies ALT. These findings provide a molecular basis underlying telomeric HR and biogenesis of C-circle and C-overhang, thus implicating the specific mechanism to resolve strand break-induced replication defect at telomeres in ALT cells.

## Author summary

10 to 15% human cancers utilize telomerase-independent alternative lengthening of telomeres (ALT) to maintain their telomere length. Unexpectedly, we find that endogenous C-strand breaks predominantly exist in telomeres of ALT cells, which induce high frequency of replication fork collapse. While collapsed fork triggers fork regression machinery to restart the replication, leading to telomeric homologous recombination; those unresolved are converted to C-circle and C-overhang. These findings suggest that the formation of C-circle and C-overhang represents a unique manner for ALT cells to prevent chromosome instability induced by replication defect at telomeres. Moreover, multiple hallmarks of ALT are provoked during this process, demonstrating that DNA strand break at telomeres underlies ALT mechanism.

## Introduction

Linear chromosome ends are capped by telomeres, which are composed of TTAGGG/CCCTAA tandem DNA repeats and a protein complex called shelterin [1–3]. Because of end replication problem [4] and possible DNA resection by Exo I (Exonuclease I) and Apollo to form single-stranded overhang [5, 6], telomeres shorten with every cell division until the critically short telomere length is reached that induces cell senescence or apoptosis [7–9]. To counteract telomere shortening, approximately 85% of human cancer cells express telomerase, while those that don’t express telomerase induce alternative lengthening of telomeres (ALT) pathway [10–12].

As a typical fragile site, telomere of ALT cells experiences a high frequency of homologous recombination (HR), which may contribute to lengthening of telomeres [13]. ALT cells are characterized by high heterogeneity of telomere length [10], an elevated frequency of telomere-sister chromatid exchanges (T-SCEs) [13, 14], the presence of APBs (ALT-associated promyelocytic leukemia nuclear bodies) [15] and abundant extrachromosomal circular telomeric DNA (C-circles) and C-rich terminal 5' overhangs (C-overhangs) [10, 16–18]. The biogenesis of C-circles and C-overhangs is not clear and their functions in cells are largely unknown. It has been proposed that telomeric DNA damage, particularly double-stranded breaks (DSBs), promotes C-circles generation in ALT cells [19, 20], and that defects in telomere replication related proteins, such as SMARCAL1 (SWI/SNF-related, matrix associated, actin-dependent, regulator of chromatin subfamily A-like 1) or the CST (CTC1/STN1/TEN1), changes the level of C-circles in ALT cells [21–23]. These results imply a potential connection between C-circles formation and DNA damage repair and/or replication defect at telomeres. Regarding to C-overhang, it appears that telomeric DNA damage is not sufficient to induce 5' C-overhangs, rather, the production of C-overhangs is associated with rapid cleavage of telomeres [24]. The question regarding whether and how the formation of C-circle and C-overhang is coordinated and their relationship with high frequency of telomeric HR and ALT remains to be elucidated.

The tandemly repeated G-rich DNA in human telomeres has a relatively high tendency to form highly compacted G-quadruplex [25]. In addition, telomeric DNA is susceptible to ultraviolet light-induced [23] and oxidative DNA damage, leading to a relatively high frequency of single- and double-stranded DNA breaks (SSBs and DSBs) and other DNA lesions in telomeric DNA [26–28]. G-quadruplex and DNA lesions frequently block replication fork progression [29–32]. In ALT cells, telomeres may experience particularly high frequency of telomeric DNA damage [17, 33, 34], leading to replication fork stalling and/or collapse. In addition, it has also been reported that ALT is linked to mutations in the ATRX/DAXX chromatin remodeling complex and histone variant H3.3, which interfere with nucleosome assembly at telomeres and likely increase replication stress [33, 35, 36]. With such replication stress, it has been interpreted that ALT cells are competent to replication defect at telomeres. Alternatively, ALT cells may develop a mechanism to cope with unsuccessfully replicated telomeres and to maintain the integrity of chromosome ends.

This study provides evidences that C-circles and C-overhangs are produced during replication of lagging and leading strand of telomeres, respectively, and that their production is associated with DNA break-induced replication fork collapse in ALT cells. Replication fork regression, which facilitates HR-dependent replication fork restart, is utilized to rescue collapsed replication fork. However, unsuccessful rescue results in the formation of C-circles and C-overhangs. Meanwhile, multiple hallmarks of ALT were raised by DNA break-induced replication fork collapse, including increased frequency of telomeric HR, formation of ALT associated PML body as well as high abundance of C-circles and C-overhangs.

## Results

### C-circles and C-overhangs arise from lagging and leading telomeric strands, respectively

**Fig 1:**
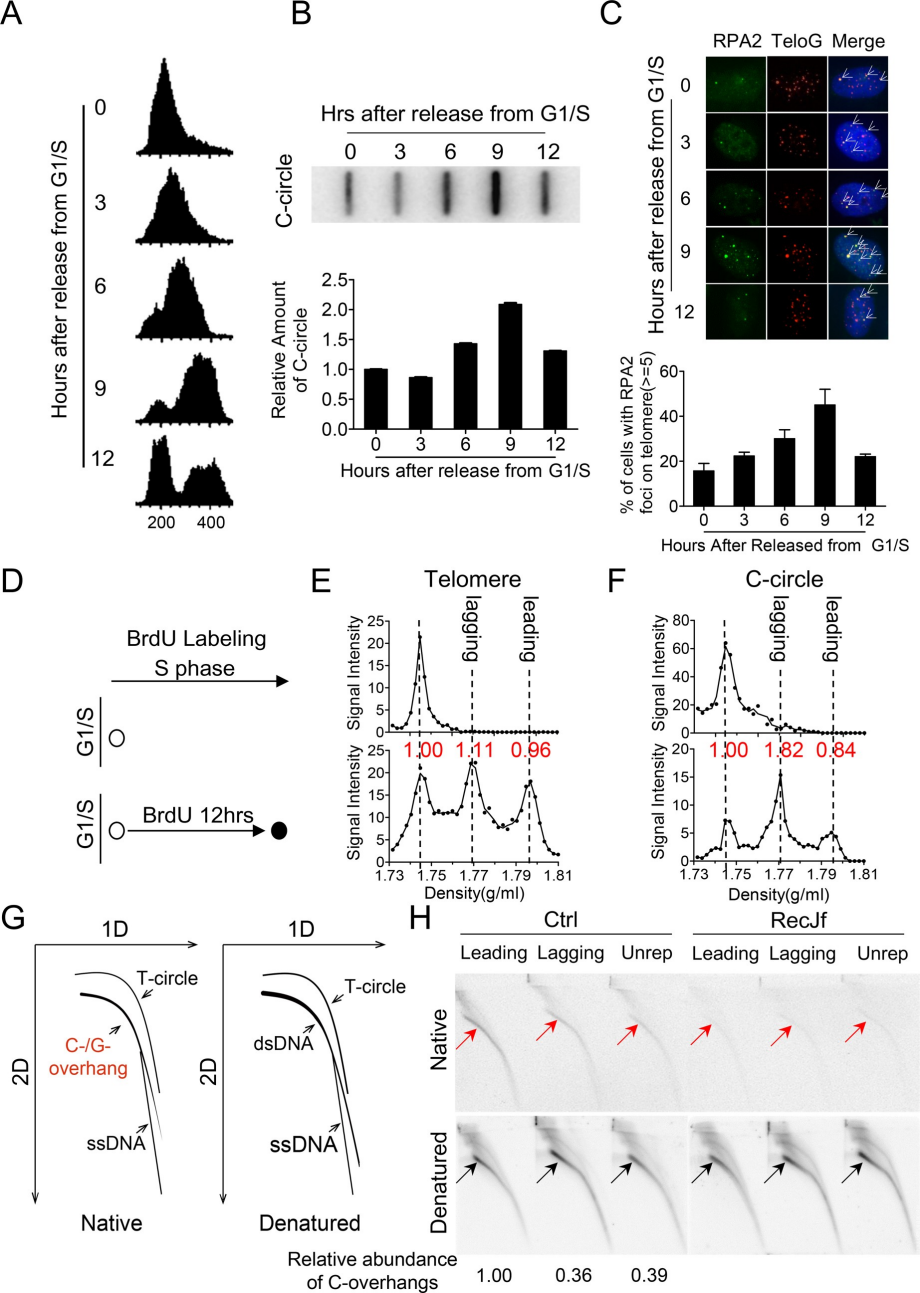
Nascent C-circles and 5' C-overhangs are generated during telomere replication. (A) FACS analysis of G1/S synchronized U2OS cells. Cells were synchronized by double thymidine block, then released and harvested at the indicated time. (B) C-circle assay was performed at the indicated time after release from G1/S. (C) Representative image and statistical analysis showing that RPA2 foci colocalize with telomere at each time point. Cells with more than 5 colocalized foci/cell were scored positively, >100 cells were counted per time point. Error bars represent the mean ± SEM of three independent experiments.(D) BrdU pulse-labeling strategy. U2OS cells were synchronized at G1/S, released in presence of BrdU for 12h. (E) Leading, lagging and unreplicated telomeric fractions were resolved by CsCl gradient ultracentrifugation and hybridized with telomeric probe. Non-BrdU labeled U2OS was used as a negative control (upper figure). “Area under peak” for leading, lagging and unreplicated telomeres was analyzed by Graphpad Prism and the relative amount of telomeres was indicated above individual peak. (F) Nascent C-circle is predominantly associated with lagging strand DNA synthesis. C-circle assay analysis of CsCl gradient fractions in (E). The amount of C-circle in leading, lagging and unreplicated telomeres was calculated by determining "area under peak" using Graphpad Prism. The relative amount of C-circles was indicated above individual peak. (G) Schematic of the migration of linear dsDNA, ssDNA (C-overhangs) and telomeric open circles (T-circle) during 2D agarose gel electrophoresis and hybridization to a telomere-specific G-rich probe under native or denatured condition. (H) 5' C-overhang DNA is predominantly associated with leading strand DNA synthesis. The fractions corresponding to leading, lagging or non-replication telomeres from 12h BrdU labeled sample in (E) were pooled. DNA was incubated with or without RecJf, analyzed by 2D agarose gel electrophoresis and hybridized with G-rich telomeric probe under native and denaturing conditions. C-overhangs were indicated by red arrows. C-overhangs abundance was expressed as a ratio between the native and denatured signals. Values were then normalized with leading C-overhangs to obtain relative abundance.

C-circle is an extrachromosomal circular telomeric DNA composed of full C-rich strand and notched G-rich complementary strand that is a quantitative biomarker of the ALT mechanism [10, 37]. Here, the cell cycle dependence of the appearance of C-circles was explored in ALT-positive U2OS cells. Specifically, U2OS cells were synchronized at G1/S by double-thymidine block, released for 0, 3, 6, 9 or 12h, corresponding to G1/S, early S, middle S, late S/G2 and G1, respectively (Fig 1A), and then assayed for the presence of C-circles. Φ29 DNA polymerase-based C-circle assay was used to determine the abundance of C-circles in cells. Reliability of method was validated by experiments in which lack of Φ29 leads to no amplified product and C-circle signal is well proportional to the amount of input DNA (R^2^ = 0.96 in linear regression of standard curve) (S1A and S1B Fig) [37]. The results showed that the abundance of C-circles increased gradually during S phase, peaked (doubled) at late S/G2 (9h after release) and decreased when cells re-entered G1 (Fig 1B). Since telomeric DNA replicates throughout S phase [38, 39], this result suggests that C-circles may be produced during telomere replication and subsequently degraded during or after G2 [40].

It has been reported that RPA2 (replication protein A2) colocalizes with telomeric DNA in human ALT cells [40, 41]. We observed that the abundance of telomeric RPA2 foci also gradually increased during S phase and decreased during or after G2 (Fig 1C). Given that RPA2 is a sensor of single-stranded DNA that might be produced during DNA damage repair and/or replication process, the specific correlation between appearance of C-circles and telomeric RPA2 foci implies that C-circle formation may associated with DNA damage response (DDR) and/or DNA replication at telomeres.

The mechanism by which C-circles form was further explored by BrdU pulse-labeling synchronized U2OS cells for 12 h after release from G1/S [42], isolating nascent C-circle and analizing its composition by CsCl density gradient ultracentrifugation (Fig 1D). The results showed that while the leading and lagging telomeric DNA was synthesized with similar efficiency (0.96 vs 1.11 in amount) (Fig 1E), BrdU-labeled C-circles were dominently enriched in lagging strand telomeric DNA (0.88 vs 1.64 for leading vs lagging synthesized C-circles after normalizing with total amount of leading or lagging telomeres) (Fig 1F), in which C-rich strand is newly synthesized and therefore BrdU-labeled. This result is consistent with the previous observation [43], the mechanism underlying the production of C-cirlce from lagging strand would be exlored below.

A similar procedure was used to determine whether 5' C-overhangs arise preferentially during leading or lagging strand DNA replication. 12h or 6h BrdU-labeled DNA was fractionated by CsCl gradient ultracentrifugation, fractions corresponding to leading, lagging and unreplicated telomeric DNA were collected, and divided into two parts, one of which was incubated with RecJf, a 5'→3' exonuclease for ssDNA, to specifically degrade 5' overhang DNA to validate telomeric C-rich ssDNA polarity [18]. The resulting samples were analyzed by neutral-neutral 2D agarose gel electrophoresis in which DNA fragments are resolved first by size in one dimension, and then by conformation in second dimension. In combination with in-gel hybridization under native or denatured conditions, 2D agarose gel electrophoresis is able to separate and distinguish linear ssDNA (G-rich or C-rich), linear dsDNA (with or without single-stranded G/C-rich overhangs) and open circular DNA (Fig 1G). C-overhangs were sensitive to RecJf digestion, but resistant to Exo I (a 3'→5' exonuclease for ssDNA), demonstrating that C-overhang is in the 5' to 3' direction (Fig 1H, S1C Fig) [18]. Both 12h and 6h BrdU labeling experiments showed that RecJf sensitive 5' C-overhangs are preferentially generated on telomeres replicated by leading strand (leading: lagging = 1.00 : 0.36 for 12h labeling sample and 1.00 : 0.29 for 6h labeling sample) (Fig 1H, S1D Fig).

### DNA strand break-induced replication fork collapse stimulates formation of C-circle and C-overhang

**Fig 2:**
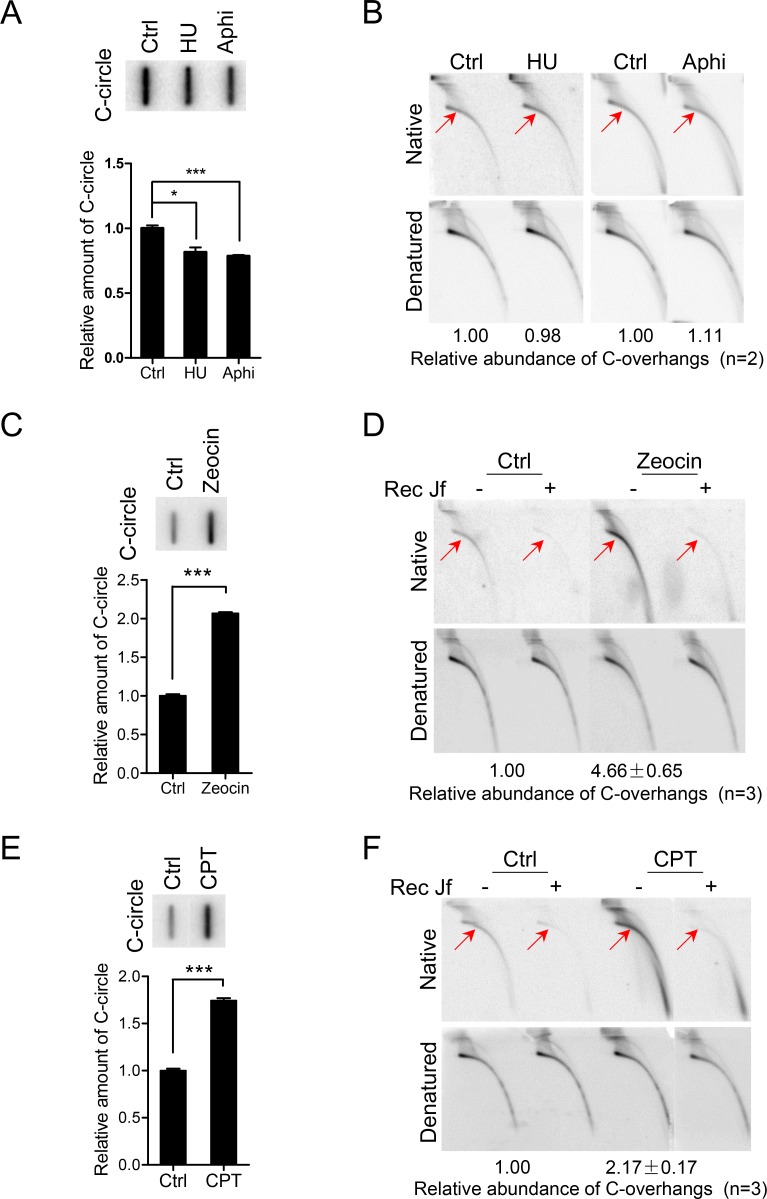
C-circles and 5' C-overhangs are linked to DNA damage-induced replication fork collapse. (A) Replication fork stalling induced by HU or aphidicolin decreases abundance of C-circles in U2OS cells. U2OS cells were treated with HU (hydroxyurea, 2mM) or aphidicolin (Aphi, 1μg/mL) for 24h and genomic DNA was purified for C-circle assay. Error bars represent the mean ± SEM of three independent experiments. Two-tailed unpaired student’s *t-*test was used to calculate P-values. *P<0.05, ***P<0.001. (B) Treatment of U2OS cells with HU or aphidicolin does not change the abundance of 5' C-overhangs. U2OS cells were treated with HU (2mM) or aphidicolin (1μg/mL) for 24h. C-overhangs abundance was expressed as a ratio between the native and denatured signals. Values were then normalized with C-overhangs in untreated cells (Ctrl) to obtain relative abundance. Experiments were duplicated and the mean of relative abundance of C-overhangs was indicated. (C) C-circles are increased in U2OS cells treated with zeocin. U2OS cells were treated with zeocin (100μg/mL) for 24h and genomic DNA was purified for C-circle assay. Error bars represent the mean ± SEM of three independent experiments. Two-tailed unpaired student’s *t*-test was used to calculate P-values. ***P<0.001. (D) 5' C-overhangs in U2OS cells, are increased upon zeocin treatment compared to DMSO. RecJf digestion was used as a control. U2OS cells were treated with zeocin (100μg/mL) for 24h. C-overhangs abundance was expressed as a ratio between the native and denatured signals. Values were then normalized with C-overhangs in untreated cells (Ctrl) to obtain relative abundance. Experiments were repeated three times and the mean ± SEM was indicated. (E) CPT (camptothecin) increases C-circles in U2OS cells. U2OS cells were treated with CPT (0.25μM) for 24h and genomic DNA was purified for C-circle assay. Error bars represent the mean ± SEM of three independent experiments. Two-tailed unpaired student’s *t*-test was used to calculate P-values. ***P<0.001. (F) CPT increases the abundance of 5' C-overhangs in U2OS cells compared to DMSO treatment. U2OS cells were treated with CPT (0.25μM) for 24h. C-overhangs abundance was expressed as a ratio between the native and denatured signals. Values were then normalized with C-overhangs in untreated cells (Ctrl) to obtain relative abundance. Experiments were repeated three times and the mean ± SEM was indicated.

To examine a potential relationship between replication-blocking and formation of C-circle and C-overhang, U2OS cells were treated with agents that result in replication fork stalling. To this end, exponentially growing U2OS cells were treated with HU (hydroxyurea) that blocks replication fork by inducing dNTP pool deficiency or aphidicolin that inhibits B-family DNA polymerase leading to replication fork stalling [44, 45]. Interestingly, both treatments resulted in no increase of RPA2 foci or DNA damage foci (p53-binding protein 1, 53BP1 foci) on telomeres (termed as TIFs: telomere dysfunction induced foci) (S2A and S2B Fig). In addition, the number of C-circles slightly decreased (Fig 2A) and the abundance of C-overhangs and G-overhangs was not significantly changed when U2OS cells were treated with HU or aphidicolin (Fig 2B, S3A Fig). These results suggested that replication fork stalling *per se* is not sufficient to stimulate the formation of C-circles and C-overhangs.

When U2OS cells were exposed to zeocin, a radio-mimetic chemical that induces oxidative DNA damage including ssDNA and dsDNA breaks [46], increased level of DDR at telomeres was detected, as expected (S2B Fig). Meanwhile, we observed increased abundance of C-circles and C-overhangs (Fig 2C and 2D). In contrast, zeocin treatment led to slight decrease of G-overhangs (S3B Fig). Importantly, we also found that the increase of C-circle and C-overhang upon zeocin treatment was restricted to S-phase (when cells were treated during S-phase), and was abrogated when cells were synchronized at G1 and exposured to zeocin (S3C–S3F Fig). Altogether, these results suggested that C-circles and C-overhangs are produced in ALT cells during telomere replication encountering DNA damages.

To imitate the situation in which replication fork progress is blocked by DNA damage, U2OS cells were treated with CPT (camptothecin), a specific inhibitor of Topo I (topoisomerase I) that induces protein-linked ssDNA break at the front of replication fork, leading to fork collapse [47, 48]. Strikingly, CPT strongly stimulated the formation of C-circles and C-overhangs in U2OS cells (Fig 2E and 2F) and the abundance of G-overhangs decreased accordingly (S3B Fig). Increased C-circle and C-overhang by zeocin or CPT treatment was also observed in other ALT VA13 cells (S3G and S3H Fig).

In a similar experiment, U2OS cells were treated with inhibitors of Topo II (topoisomerase II), VP-16 (etoposide) or ICRF-187 (dexrazoxane) [49–51]. VP-16 induces protein-linked dsDNA breaks in replicating DNA leading to replication fork collapse, while ICRF-187 inhibits the cleavage activity of Topo II leading to replication fork stalling [52]. Interestingly, VP-16 stimulated formation of C-circles and C-overhangs, whereas the abundance of G-overhangs decreased. However, ICRF-187 treatment showed a limited effect on C-circle, C-overhang and G-overhangs (S4A–S4C Fig). This result further demonstrated that DNA damage-induced replication fork collapse rather than replication fork stalling promotes formation of C-circles and C-overhangs. This conclusion was further confirmed by duplicating the experiments in other ALT positive VA13 cells (S4D–S4F Fig).

### Intrinsic DNA damage at C-rich strand of telomeres leads to the formation of C-circle and C-overhang

**Fig 3:**
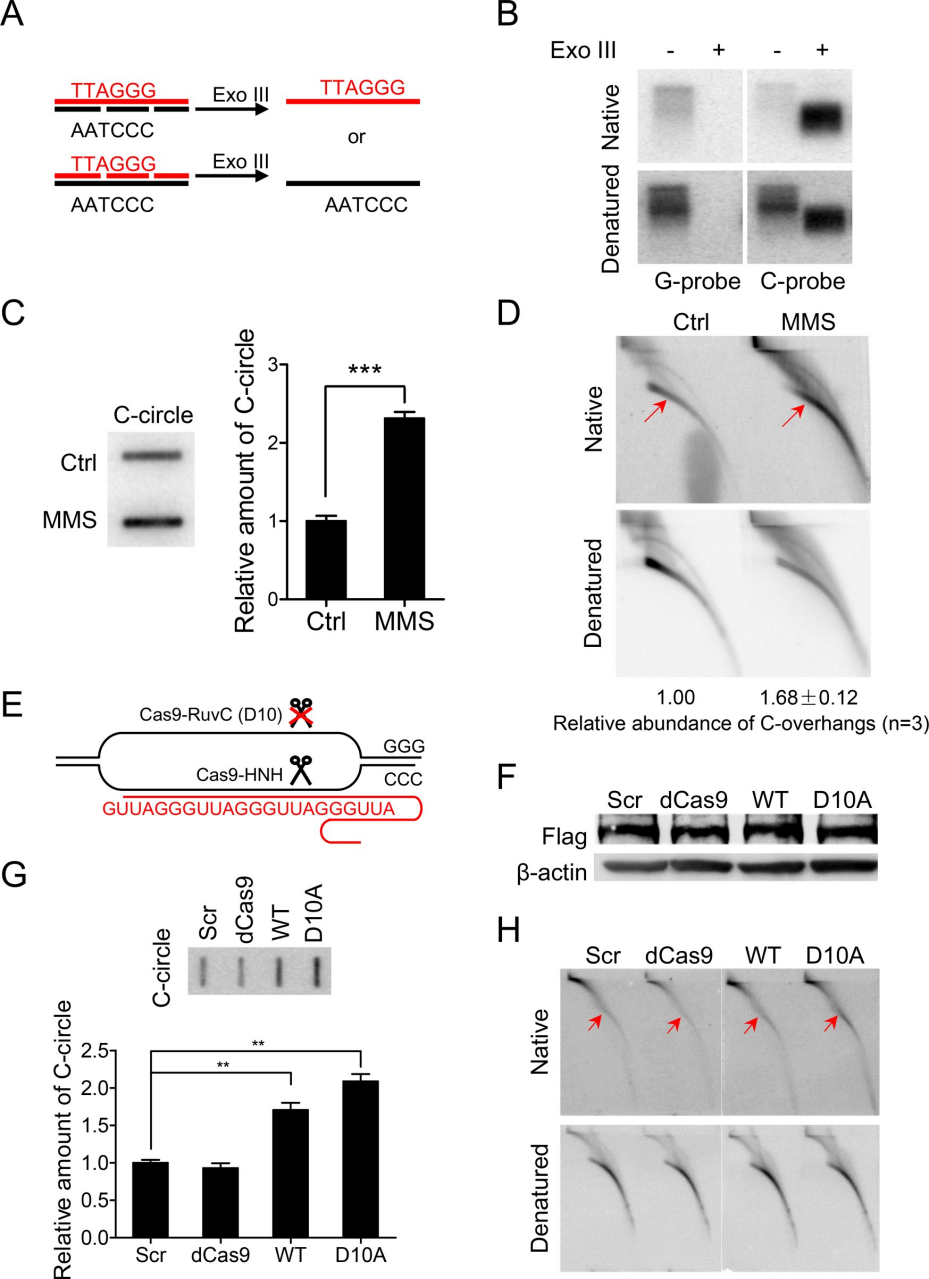
Endogenous ssDNA break/gap or induced ssDNA break in C-rich strand stimulates formation of C-circles and 5' C-overhangs. (A) Experimental protocol to study strand specific (G-rich or C-rich) breaks/gaps on telomere is shown schematically. HinfI and RsaI digested genomic DNA was purified and further digested with Exo III to examine potential breaks/gaps on G-strand or C-strand of telomeres. If breaks/gaps occur on C-strand, Exo III would degrade all C-strand, leaving single-stranded G-strand that can be detected by hybridization with C-rich probe under native or denatured condition. Contrariwise, only C-strand can be detected if breaks/gaps occur on G-strand. (B) Breaks/gaps occur more frequently on C-rich strand of telomere. Exo III digestion produces single-stranded DNA that is less in molecular weight than corresponding double-stranded DNA, thereby migrating faster during electrophoresis. (C) Methyl-methane sulfonate (MMS) stimulates formation of C-circle DNA in U2OS cells. U2OS cells were treated with MMS (0.25mM) for 24h and genomic DNA was purified for C-circle assay. Error bars represent the mean ± SEM of three independent experiments. Two-tailed unpaired student’s *t*-test was used to calculate P-values. ***P<0.001. (D) MMS stimulates formation 5' C-overhang DNA in U2OS cells. U2OS cells were treated with MMS (0.25mM) for 24h. C-overhangs abundance was expressed as a ratio between the native and denatured signals. Values were then normalized with C-overhangs in untreated cells (Ctrl) to obtain relative abundance. Experiments were repeated three times and the mean ± SEM was indicated. (E) Experimental protocol using CRISPR-Cas9 system to introduce ssDNA breaks at telomere is shown schematically. Cells express nuclease-deficient CRISPR-Cas9 (dCas9), wild type CRISPR-Cas9 (WT) or CRISPR-Cas9 with mutation at RuvC domain (D10A). dCas9 lacks nuclease activity, wtCas9 introduces dsDNA breaks, and Cas9 D10A introduces ssDNA breaks in C-rich strand of telomere. (F) Western blot of dCas9, wtCas9and Cas9 D10A expressed in HEK 293T cells. Cells are harvested 48h after transfection. Monoclonal ANTI-FLAG M2 antibody was used to determine expression level of flag-Cas9. β-actin was used as a loading control. (G) Effect of WT and mutant Cas9 on formation of C-circles in HEK 293T cells. Error bars represent the mean ± SEM of three independent experiments. Two-tailed unpaired student’s *t*-test was used to calculate P-values. **P<0.01. (H) Effect of WT and mutant Cas9 on formation of 5' C-overhangs in HEK 293T cells.

Given a high abundance of C-circles and C-overhangs in ALT cells, we then asked whether intrinsic DNA strand breaks exist in telomeres that induce replication fork collapse. For this purpose, genomic DNA was digested with HinfI and RsaI, followed by digestion with Exo III (Exonuclease III), a 3' to 5' exonuclease that remove nucleotides from blunt end or break/gap in double stranded DNA to generate single stranded DNA (Fig 3A). The digested DNA were hybridized with C-rich or G-rich probe under native or denatured condition [53] (Fig 3A). The rationale was to determine whether endogenous ssDNA breaks and gaps occur in the G-rich or C-rich strand of telomeric DNA in ALT cells. If such lesions were enriched in the C-rich strand, it would be preferentially degraded by Exo III, leaving primarily G-rich ssDNA to hybridize with a C-rich probe, while the reverse specificity, or lack of specificity would be observed in the absence of preferential endogenous ssDNA breaks in the C-rich strand of the telomere (Fig 3A). We detected much more G-rich ssDNA than C-rich ssDNA (Fig 3B). This suggests that endogenous ssDNA breaks and gaps are present predominantly on the C-rich strand of telomeric DNA in U2OS cells.

MMS (Methyl-methanesulfonate) is a DNA damaging agent that preferentially creates mutagenic lesions in cytosine of ssDNA [54, 55]. Here, we showed that exposure to MMS significantly stimulates formation of C-circles and C-overhangs in ALT cells (Fig 3C and 3D). To further confirm that ssDNA breaks in the C-rich strand of telomeric DNA induce formation of C-circle and C-overhang, we expressed CRISPR-Cas9 with a D10A mutation in the RuvC nuclease domain of Cas9 (Cas9-D10A), which specifically generates ssDNA breaks in the strand complementary to sgRNA (Fig 3E) [56, 57]. Indeed, when sgRNA with telomeric G-rich sequence (sgTel) was co-expressed with Cas9-D10A in U2OS cells, we observed significant number of C-rich, but not G-rich DNA fragments (smear on gel) that are released from telomeres and detected by alkaline constant-field gel electrophoresis (alkaline plug assay, see Method for detail) (S5A and S5B Fig), indicating specific induction of DNA breaks by Cas9-D10A at C-rich strands. As expected, expression of wild-type Cas9 (wtCas9, WT) induced double-stranded breaks at telomeres [58], leading to increase of both G- and C-rich fragments (S5A and S5B Fig). However, expression of nuclease-deficient mutant Cas9 (dCas9) led to no increase of G- or C-rich fragments (S5A and S5B Fig). We observed significant increase of C-circles and C-overhangs in cells expressing wtCas9 or Cas9-D10A but not in cells expressing dCas9 (S5C and S5D Fig). Similar experiments were also performed in non-ALT human HEK 293T cells. Western blot analysis demonstrated that wtCas9, dCas9 and Cas9-D10A protein were expressed at a similar level (Fig 3F). The results showed that expression of both wtCas9 and Cas9-D10A stimulated formation of C-circles (Fig 3G). The formation of C-overhangs was strongly stimulated by expression of Cas9-D10A (Fig 3H). And the expression of wtCas9 also slightly increased the abundance of C-overhangs, consistent with our previous finding that telomeric DSB initiates homologous recombination mediated repair that produces 3′ C-rich overhang [58]. Collectively, these results suggest that endogenous breaks/gaps or extraneously induced ssDNA breaks in C-rich strand of telomeric DNA stimulates formation of C-circles and C-overhangs.

### Mechanism underlying rescue of DNA damage-induced replication fork collapse at telomeres

**Fig 4:**
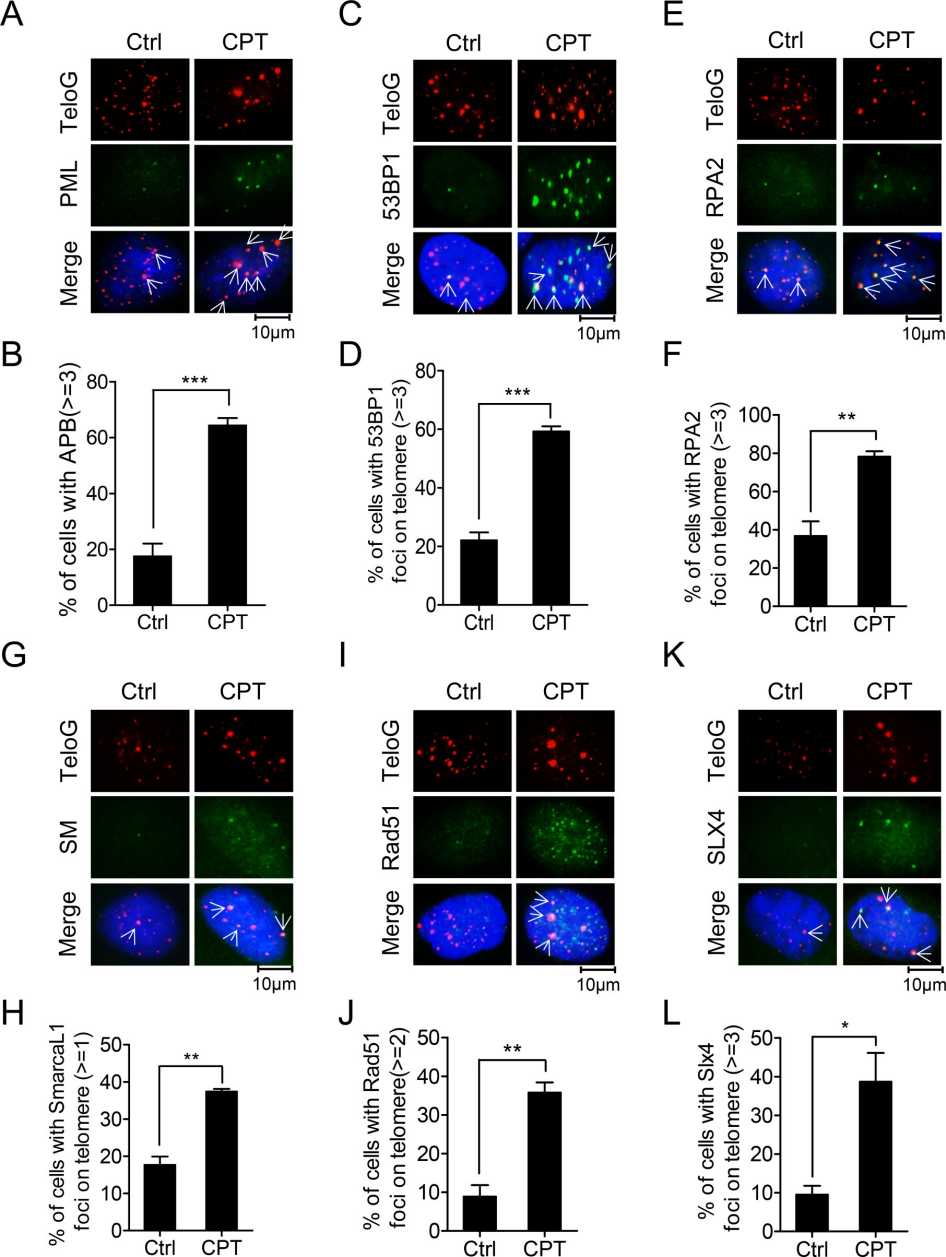
Replication fork collapse and replication fork regression at telomeres in U2OS cells. (A) U2OS cells were cultured in presence or absence of CPT and analyzed with IF-FISH to detect PML bodies on telomeres. (B) Quantification of (A). Cells with ≥3APBs were scored. >100 cells were counted for each experiment. Error bars represent the mean ± SEM of three independent experiments. Two-tailed unpaired student’s *t*-test was used to calculate P-values. ***P<0.001. (C) U2OS cells were treated with or without CPT and analyzed by IF-FISH to detect 53BP1 foci on telomeres. (D) Quantification of (C). Cells with ≥3 co-stained foci were scored. >100 cells were counted for each experiment. Error bars represent the mean ± SEM of three independent experiments. Two-tailed unpaired student’s *t*-test was used to calculate P-values. ***P<0.001. (E)-(L) U2OS cells were treated with or without CPT and analyzed by IF-FISH using telomeric G-rich probe and antibodies to RPA2 (E), SMARCAL1 (G, SM), Rad51 (I) or SLX4 (K), respectively. Quantification of panels (E), (G), (I), and (K) are shown in (F), (H), (J), and (L), respectively. Cells with ≥3 RPA2 (F), ≥1 SMARCAL1 (H),≥2 Rad51 (J), ≥3 SLX4 (L) foci colocalized with telomeres were scored. >100 cells were counted for each experiment. Error bars represent the mean ± SEM of three independent experiments. Two-tailed unpaired student’s *t*-test was used to calculate P-values. *P<0.05, **P<0.01.

To explore how ALT cells respond to replication fork collapse, CPT-treated U2OS cells were analyzed using IF-FISH (immunofluorescence and fluorescence *in situ* hybridization) to determine the proteins enriched at telomeres in response to replication fork collapse induced by CPT treatment. Strikingly, the abundance of PML foci and ALT associated PML bodies (APBs) increased in cells exposed to CPT (Fig 4A and 4B). In addition, we observed a significant increase of 53BP1 foci genome-wide and at telomeres when cells were treated with CPT (Fig 4C and 4D). Moreover, RPA2 foci at telomeres increased in CPT treated cells (Fig 4E and 4F). We also found that SMARCAL1, Rad51 and SLX4 were recruited to telomeres in U2OS cells and their foci at telomeres were significantly increased when cells were challenged with CPT (Fig 4G–4L). Importantly, RPA2/Rad51/SMARCAL1/SLX4 compose a machinery termed replication fork regression [21, 31, 59–61], a mechanism that rescues collapsed replication fork.

### Replication fork regression prohibits formation of C-circles and C-overhangs

**Fig 5:**
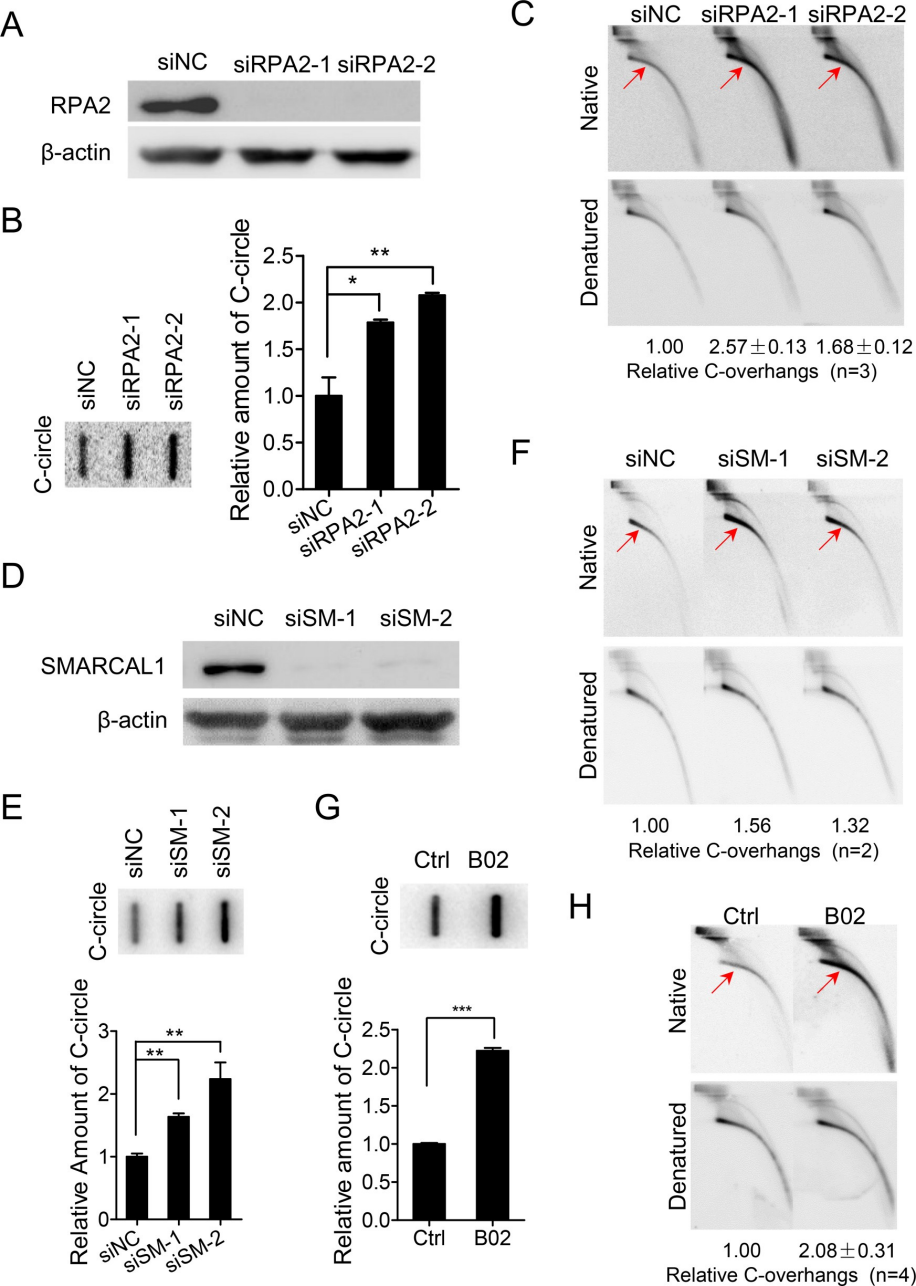
Defect in replication fork regression increases abundance of C-circles and 5' C-overhangs. (A) Western blot shows efficiency of RPA2 knockdown by siRNA. β-actin was used as a loading control. U2OS cells were collected 60h after transfection with siRNA. (B) Abundance of C-circles in RPA2-depleted cells. Error bars represent the mean ± SEM of three independent experiments. Two-tailed unpaired student’s *t*-test was used to calculate P-values. *P<0.05, **P<0.01. (C) Abundance of 5' C-overhangs in RPA2-depleted cells. C-overhangs abundance was expressed as a ratio between the native and denatured signals. Values were then normalized with C-overhangs in control cells (siNC) to obtain relative abundance. Experiments were repeated three times and the mean ± SEM was indicated. (D) Western blot shows efficiency of SMARCAL1 (SM) knockdown by siRNA. β-actin was used as a loading control. U2OS cells were collected 60h after transfection with siRNA. (E) Abundance of C-circles in SMARCAL1-depleted cells. Error bars represent the mean ± SEM of three independent experiments. Two-tailed unpaired student’s *t*-test was used to calculate P-values. **P<0.01. (F) Abundance of 5' C-overhangs in SMARCAL1-depleted cells. C-overhangs abundance was expressed as a ratio between the native and denatured signals. Values were then normalized with C-overhangs in control cells (siNC) to obtain relative abundance. Experiments were duplicated and the mean of relative abundance of C-overhangs was indicated. (G) Effects of Rad51 inhibitor B02 on abundance of C-circles. U2OS cells were treated with B02 (27.4μM) for 24h. Error bars represent the mean ± SEM of three independent experiments. Two-tailed unpaired student’s *t*-test was used to calculate P-values. ***P<0.001. (H) Effects of Rad51 inhibitor B02 on abundance of 5' C-overhangs. C-overhangs abundance was expressed as a ratio between the native and denatured signals. Values were then normalized with C-overhangs in control cells (siNC) to obtain relative abundance. Experiments were repeated four times and the mean ± SEM was indicated.

Evidences presented above suggest that replication fork collapse in telomeric DNA is tightly linked to formation of C-circle and C-overhang structures. Therefore, it was predicted that replication fork regression, mediated by RPA2-Rad51-SMARCAL1-SLX4 axis, might rescue collapsed replication fork and thus suppress formation of C-circles and C-overhangs. To test this, U2OS cells were treated with RPA2- or SMARCAL1-targeted siRNA and the abundance of C-circles and C-overhangs was examined (Fig 5A and 5D). Indeed, C-circles and C-overhangs were more abundant in RPA2 or SMARCAL1-deficient U2OS cells (Fig 5B, 5C, 5E and 5F). Accordingly, G-overhangs were slightly decreased (S6A Fig). Moreover, when Rad51 was inhibited by B02, a specific inhibitor of Rad51 [62], the abundance of C-circles and C-overhangs also increased, while G-overhangs decreased (Fig 5G and 5H, S6B Fig). These results suggested that replication fork regression prevents the formation of C-circles and C-overhangs in U2OS cells. The same experiments were also repeated in VA13 cells. Consistently, we observed that both depletion of RPA2 (or SMARCAL1) and inhibition of Rad51 by B02 stimulated formation of C-circles and C-overhangs, but reduced the abundance of G-overhangs (S6C–S6I Fig).

Meanwhile, we observed that pDNA-PKcs (DNA-dependent protein kinase, catalytic subunit), a key sensor in the NHEJ (non-homologous end-joining) pathway, localizes to telomeres in U2OS cells, and that telomeric pDNA-PKcs (S2056) foci are more abundant in CPT-treated cells (S7A and S7B Fig). To explore whether NHEJ plays a role in production of C-circles or C-overhangs, U2OS cells were treated with DNA-PKcs inhibitor NU7441. Previous report demonstrated that inhibition of ATR by VE-821 leads to decrease of C-circles [23]. Our results showed that NU7441 treatment decreased the level of C-circles (S7C Fig), whereas the abundance of C-overhangs and G-overhangs remained largely unchanged (S7D and S7E Fig). These findings implied that NHEJ machinery promotes circularization of lagging strand DNA at collapsed telomeric replication fork, thus enabling formation of C-circles.

### Strand-break induced replication fork collapse is linked to telomeric HR

**Fig 6:**
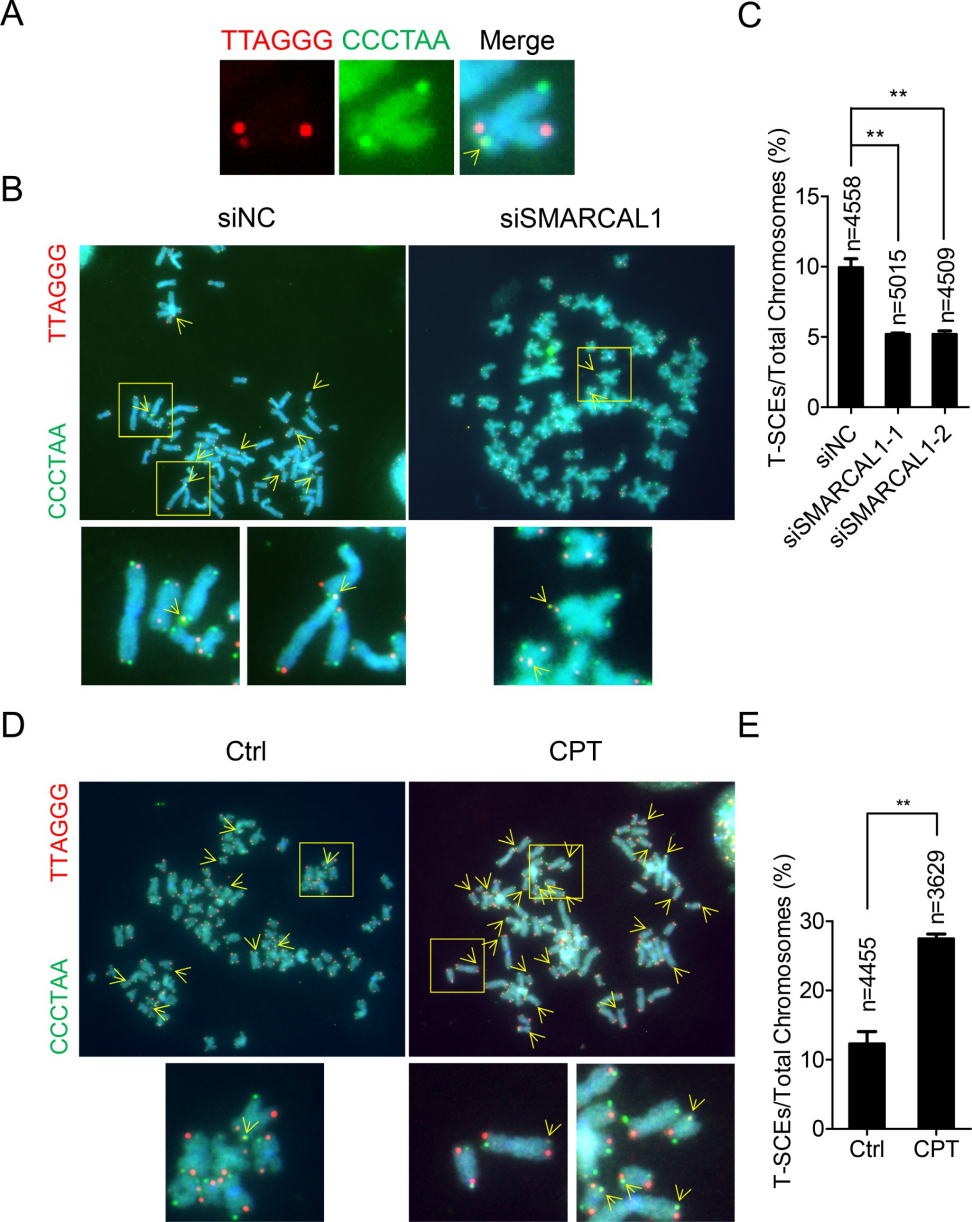
Telomeric HR is associated with replication fork regression. (A) Representative images showing C-strand (red) and G-strand (green) of telomeres on sister chromatins that are visualized by CO-FISH (chromosome orientation fluorescence *in situ* hybridization) assay. Yellow spot representing the occurrence of T-SCE was indicated by yellow arrows. (B) Representative images showing T-SCEs in U2OS cells depleted for SMARCAL1. (C) Quantification of (B). The number of chromosomes scored (n) in three independent experiments is indicated. Error bars represent the mean ± SEM of three independent experiments. Two-tailed unpaired student’s *t*-test was used to calculate P-values. **P<0.01. (D) Representative images showing T-SCEs in U2OS cells treated with CPT. (E) Quantification of (D). The number of chromosomes scored (n) in three independent experiments is indicated. Error bars represent the mean ± SEM of three independent experiments. Two-tailed unpaired student’s *t*-test was used to calculate P-values. **P<0.01.

Previous studies suggest that replication fork regression is coupled with HR to reinitiate replication [19, 31, 61]. Consistent with this, we observed that telomere sister chromatid exchange (T-SCE), which indicates HR occurring at telomeres, was inhibited (i.e., reduced frequency) in SMARCAL1-knockdown U2OS cells (Fig 6B and 6C). In addition, when replication fork collapse was induced by CPT treatment in U2OS cells, we observed increased frequency of T-SCE (Fig 6D and 6E). These results supported the hypothesis that replication fork collapse at telomeres, which is rescued by replication fork regression-mediated process, leads to telomeric recombination.

Rad51 plays a key role in both replication fork regression and telomeric recombination [19, 31]. When U2OS cells were treated with B02, a specific inhibitor of Rad51 [62], telomeric PCNA and RPA2 foci increased (S8A–S8D Fig), likely due to the accumulation of collapsed replication fork [50]. Consistently, less fully synthesized telomeric DNA were detected (S8E Fig). After treatment with B02 for 4 days, short telomeres were accumulated in U2OS cells (S8F Fig).

## Discussion

This study investigates the biogenesis of C-circles and C-overhangs in ALT cells. Evidence is presented that C-circles and C-overhangs represent circularized lagging and broken leading strands of telomeric DNA, respectively, and that their formation is tightly linked to strand break-induced collapse of DNA replication forks in telomeric DNA in ALT cells. Although replication fork regression and HR-mediated replication restart can rescue replication fork collapse, the formation of C-circle and C-overhang on unresolved replication fork may represent a new manner for ALT cells to cope with unsuccessfully replicated telomeres and to maintain chromosome integrity.

### Breaks in the C-Rich strand stimulate formation of C-circles and C-overhangs

**Fig 7:**
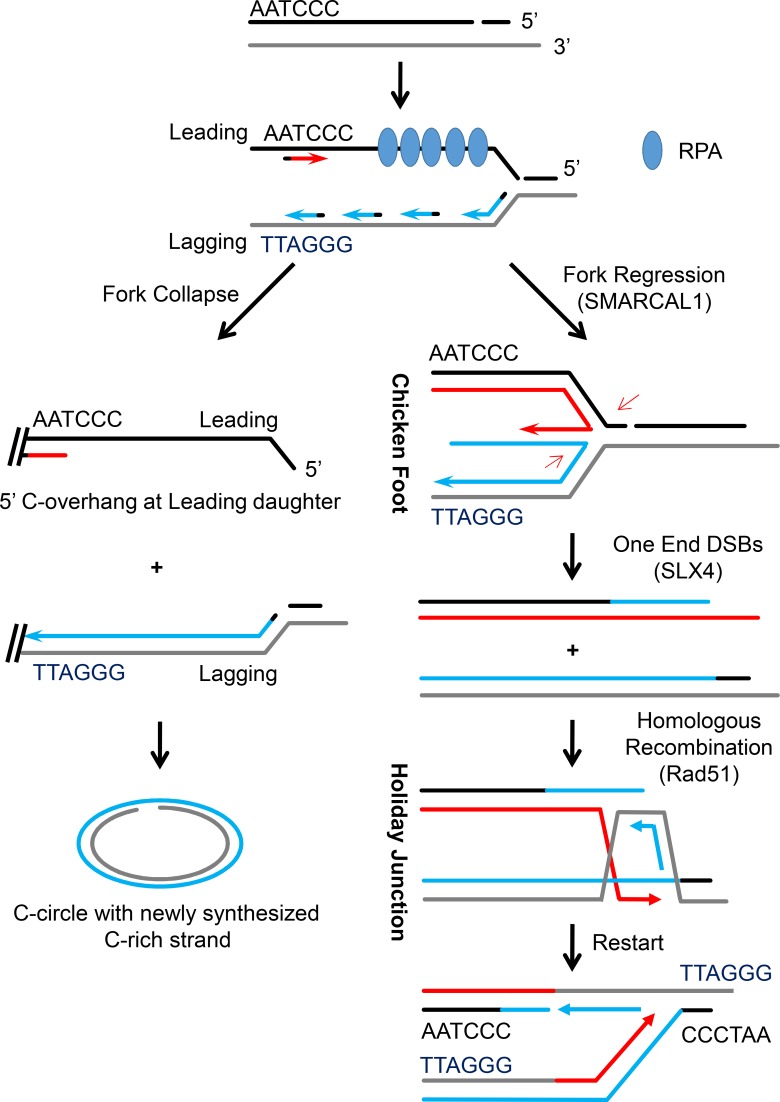
Proposed model summarizing effects of replication fork collapse and replication fork regression on formation of C-circles and C-overhangs in ALT cells. Replication fork collapse induced by break or gap on the C-rich strand of telomere, if not rescued, leads to different consequences for leading and lagging replication: for leading synthesis, long single-stranded C-rich DNA (C-overhang) remains unreplicated, whereas stalled lagging replication fork is cut out and cyclized to form C-circles. Fork regression machinary including SMARCAL1, SLX4 and Rad51 may restore collapsed replication fork, and thereby suppressing C-circle and C-overhang formation. HR-mediated fork regression is based on the model proposed by Petermann and Helleday [31].

The results show that replication fork collapse leading to C-circles and C-overhangs can be induced by exogenous agents that generate ssDNA or dsDNA breaks in telomeric DNA (zeocin, CPT, VP16, MMS and CRISPR-Cas9 system). In addition, we found that endogenous lesions occur preferentially in the C-rich strand of telomeres in ALT cells (Fig 3B). During replication progression, the C-rich strand templates leading strand DNA synthesis, while the G-rich strand templates lagging strand DNA synthesis [63]. Replication fork collapse, induced by a break or gap on the C-rich strand, has different consequences for leading and lagging replication (Fig 7). For leading synthesis, long single-stranded C-rich DNA remains unreplicated, forming C-overhangs at the end of the chromosome; lagging strand synthesis still proceeds, but would likely lead to “looping-out” during which the stalled replication fork is cut out and cyclized to form C-circles [50]. The evidence supporting this model include: 1) C-circles are primarily derived from the lagging strand, whereas C-overhangs are primarily derived from the leading strand (Fig 1); 2) replication fork collapse, but not conventional replication fork stalling, stimulates production of C-circles and C-overhangs (Fig 2); 3) endogenous breaks/gaps are present in the C-rich telomeric strand and agents that introduce ssDNA breaks in the C-rich telomeric strand (i.e., MMS treatment and CRISPR-Cas9 (D10A)) stimulate formation of C-circles and C-overhangs (Fig 3); 4) the formation of C-circles is NHEJ-dependent (S7 Fig).

### Unsolved replication fork collapse leads to formation of C-circle and C-overhang

Spontaneous telomeric DNA damage response and telomeric RPA2 foci are often observed in ALT cells [40, 64]. We found that RPA2 accumulate in telomeric DNA of CPT-treated cells (Fig 4E and 4F) but not in HU- or aphidicolin-treated cells (S2A Fig), suggesting that replication fork collapse, but not replication fork stalling, is the signal that recruits RPA2. Binding of RPA2 to ssDNA activates the ATR pathway, leading to replication fork protection and restoration [23, 65]. DNA damage-induced replication fork collapse can be rescued by replication fork regression [48]. And, SMARCAL1, which is activated by ATR-dependent phosphorylation, plays an essential role in replication fork regression [66]. Indeed, it has been previously reported that the deficiency of SMARCAL1 leads to fragile telomeres [60]. Our results also demonstrated that components of fork regression machinery including Rad51, SMARCAL1 and SLX4 are recruited to telomeres in CPT-treated cells (Fig 4E–4L). This, however, does not exclude the possibility that the mechinary other than fork regression might also be adopted to rescue collapsed replication fork. In fact, it has been reported that translesion synthesis, which is composed of FANCJ, RAD18, ^ub^PCNA and Polη, is engaged in bypassing DNA lesions on replication fork [67] [68].

Interestingly, depletion or inhibition of each component of fork regression machinery stimulated formation of C-circles and C-overhangs (Fig 5). We thus proposed that endogenous breaks/gaps in C-rich strand of telomeric DNA in ALT cells either induce replication fork collapse, leading to C-circle and C-overhang structures, or, fork regression or other fork rescue machinary restores the collapsed replication fork, and C-circle and C-overhang formation is suppressed (Fig 7). In supporting this model, it has been recently discovered that SMARCAL1 loss-of-function mutations in cancers link to the ALT mechanism of telomere maintenance, resulting in ultrabright telomeric foci and the generation of C-circles [69]. In additon, deficiency of Polη, which is essential for translesion synthesis, increases replication stress at telomeres and stimulates the formation of C-circles [68].

### Replication fork collapse and ALT mechanism

ALT cells are characterized by high frequency telomeric recombination [13]. It has been found that a DNA damage at ALT telomeres triggers long-range movement and clustering, resulting in homology-directed telomere synthesis between sister and non-sister chromatids [19, 70]. Here, we demonstrated that DNA strand break in the C-rich strand of the telomere leads to replication fork collapse, followed by replication fork regression and telomeric HR (Fig 6). Whether or not this recombination contributes to telomere elongation remains to be elucidated. However, homologous searching and recombination could occur anywhere along the telomere due to its repetitive nature, creating the possibility for telomere extension (Fig 7). In the presence of Rad51 inhibitor B02, short telomeres accumulate (S8F Fig), supporting the idea that Rad51-dependent HR promotes telomere extension. Furthermore, it is worth noting that APBs accumulate in CPT-treated ALT cells (Fig 4). Therefore, replication fork collapse provokes multiple hallmarks of ALT, including telomeric HR, APBs, C-circles and C-overhangs. While C-circles and C-overhangs are associated with telomere trimming, telomeric HR might contribute to telomere elongation. Additional studies are needed to understand how these events are coordinated in order to maintain chromosome end integrity and telomere length homeostasis in ALT cells.

## Material and methods

### Cell culture and treatment

U2OS, HEK 293T and VA13 cells were grown in Dulbecco’s modified Eagles’ medium (DMEM) supplemented with 10% fetal bovine serum, 1% penicillin/streptomycin at 37°C in 5% CO_2_. Unless otherwise indicated, cell lines were treated with HU (2mM, Sigma) or aphidicolin (1μg/mL, Sigma) or zeocin (100μg/mL, Thermo Fisher) or CPT (0.25μM, MCE) or MMS (0.25mM, Sigma) or VP-16 (10μM, Sigma) or ICRF-187 (50μg/mL, Selleck) or B02 (27.4μM, EMD Millipore) or VE821 (10μM, MCE) or NU7441 (250nM, Selleck). Knockdown experiments were performed with the Lipofectamine RNAiMAX Reagent (Thermo Fisher Scientific) using following siRNA targets:

siNC/UUCUCCGAACGUGUCACGUdTdT/ACGUGACACGUUCGGAGAAdTdT; siRPA2-1/GGCUCCAACCAACAUUGUUdTdT/AACAAUGUUGGUUGGAGCCdTdT; siRPA2-2/GCCUGGUAGCCUUUAAGAUdTdT/AUCUUAAAGGCUACCAGGCdTdT; siSMARCAL1-1/CCAAGAGACACCAGCUCAUdTdT/

AUGAGCUGGUGUCUCUUGG dTdT;

siSMARCAL1-2/UUGCUAAGAAGGUCAAAGCdTdT/

GCUUUGACCUUCUUAGCAAdTdT. Cells were collected 60h after transfection for experiments.

### Plasmid construction

Lenti-CRISPRv2 (Addgene plasmid # 52961) was used in this study [71]. Scaffold sequence of sgRNA was modified to

5'-NNNNNNNNNNNNNNNNNNGUUUAAGAGCUAUGCUGGAAACAGCAUA GCAAGUUUAAAUAAGGCUAGUCCGUUAUCAACUUGAAAAAGUGGCACCGAGUCGGUGCUUUUUUU-3′, as previously described [58, 72]. sgSCR (5′-TGCTCCGTGCATCTGGCATC-3'), sgTel (5'-GTTAGGGTTAGGG TTAGGGTTA-3') [58] were cloned as described previously [73]. Cas9 mutations, including D10A and dead-nuclease (D10A, H840A), were constructed by mutagenesis kit (Fast Mutagenesis System, Transgen Biotech). The transfection was carried out with Lipofectamine 2000 Reagent (Thermo Fisher Scientific).

### Cell cycle synchronization and BrdU labeling

U2OS cells were synchronized at G1/S by "double thymidine block" method as described previously [42]. Briefly, exponentially growing U2OS cells were blocked with 2mM thymidine for 19h, washed three times with prewarmed PBS and released into fresh medium for 10h, and then blocked again with 2mM thymidine for another 14h. For 5-bromo-2-deoxyuridine (BrdU, Sigma) labeling, cells were incubated with fresh medium containing 100μM BrdU for 12 h after release from G1/S. FACS analysis was carried out as described previously [42].

### Genomic DNA purification and enzyme digestion

All genomic DNA was extracted and purified using AxyPrep Blood Genomic DNA Miniprep Kit (Axygen) according to manufacturer’s instructions. DNA concentration was measured by Nanodrop-2000. For 2D agarose gel analysis, 10μg DNA was digested overnight at 37°C with 10U HinfI (Thermo Fisher), 10U RsaI (Thermo Fisher) and 2μg/mL RNase A (Takara). The reaction was terminated with EDTA and analyzed by 2D agarose gel electrophoresis. 30U RecJf (New England Biolabs) was added for removing 5' single-stranded DNA.

For internal gaps/nicks analysis, 5μg genomic DNA was digested overnight at 37°C with 5U HinfI (Thermo Fisher), 5U RsaI (Thermo Fisher) and 1μg/mL Ribonuclease A (RNase A, Takara) and purified with QIAquick PCR Purification kit (Qiagen). Purified DNA were digested with or without 200U Exonuclease III (New England Biolabs) overnight at 37°C, and then subjected to 0.7% agarose gel electrophoresis and in gel hybridization.

### CsCl gradient ultracentrifugation

CsCl gradient ultracentrifugation and DNA purification were performed as described previously [5, 42]. DNA was purified and dissolved in 60μL ddH2O. One half of each sample was incubated with RecJf prior to analysis by 2D agarose gel electrophoresis.

### C-circle assay

C-circle assay was performed as described previously [37]. The concentration of genomic DNA was determined by fluoremetry based method (Qubit 3.0 Fluorometer, Thermo Fisher Scientific). Exactly the same amount of genomic DNA was input for C-circle assay (30ng for U2OS, 100ng for HEK 293T and VA13 cells). Each assay was repeated three times to obtain the quantitative result. To determine C-circles in CsCl fractions, 1μL of each fraction was incubated in 40μL reaction containing 19μL ddH2O and 20μL C-circle amplification master buffer (0.2mg/mL BSA, 0.1% Tween 20, 1mM dATP, dGTP and dTTP each, 1× Φ29 Buffer and 7.5U Φ29 DNA polymerase (Thermo Fisher)) for 8h at 30°C, and then subjected to slot-blot and hybridization with C-probe.

### Neutral-Neutral two-dimensional gel electrophoresis

Neutral-Neutral 2D agarose gel electrophoresis was performed as described previously [74, 75]. Briefly, enzyme digested DNA samples were loaded into a 0.4% agarose gel for first dimension and electrophoresis was performed at 1V/cm for 12 h at room temperature in TBE buffer. Lanes were excised, soaked in TBE containing 0.3μg/mL ethidium bromide (EB) (Sigma) for 30min, embedded in 1% agarose gel containing EB. Second dimension electrophoresis was performed at 4°Cfor 6 h at 3V/cm. The gel was then dried at room temperature by vacuum drier, and hybridized with G-/C-probe under native or denatured condition.

### Telomere restricted fragment (TRF) assay

The telomere length assay was performed as previously described [42]. 5μg genomic DNA was digested overnight at 37°C with 5U HinfI (Thermo Fisher), 5U RsaI (Thermo Fisher) and 1μg/mL RNase A (Takara). Digested DNA samples were subjected to conventional 0.7% agarose gel in TAE buffer at 2V/cm for 16h at room temperature. The gel was dried at 42°C with vacuum drier, and hybridized with C-probe.

### Immunofluorescence-fluorescence *in situ* hybridization (IF-FISH)

IF-FISH was performed as previously described [50, 58].

Fluorescent probe is Cy3-(TTAGGG)_3_ (Panagene). Primary antibodies include anti-53BP1 (Novus Biologicals), anti-RPA2 (EMD Millipore), anti-PCNA (Genetex), anti-PML (Santa Cruz), anti-SMARCAL1 (Santa Cruz), anti-SLX4 (Novus Biologicals), anti-pDNA-PKcs (S2056) (Abcam) and anti-Rad51 (Santa Cruz). Secondary antibodies include DyLight488 conjugated anti-rabbit (Multisciences), DyLight488 conjugated anti-mouse (Multisciences).

### Chromosome orientation fluorescence *in situ* hybridization (CO-FISH)

The telomeric sister chromatid exchange (T-SCE) was determined by CO-FISH, which is performed as described previously [58, 76]. Fluorescent probes are Cy3-(TTAGGG)_3_ (Panagene) and FITC-(CCTAAA)_3_ (Panagene).

### Alkaline constant-field gel electrophoresis (alkaline plug assay)

The alkaline constant-field gel electrophoresis was performed as described previously described [77]. Briefly, 1×10^6^ cells were rinsed with 1×PBS, resuspended in 50μl 0.7% 45°C pre-warmed agarose (made with 1×TE, pH 8.0), and solidified in 1ml decapitated injector. Agarose plugs were incubated in fresh-made lysis-buffer (30mM Tris-HCl pH8.0, 300mM NaCl, 25mM EDTA, 0.5% SDS, 0.1mg/ml Protease K, 0.1mg/ml RNase A) overnight. The plugs were then denatured in 100mM NaOH with 1mM EDTA, placed into the wells of 0.7% alkaline agarose gel (50mM NaOH with 1mM EDTA) and sealed with 0.7% alkaline agarose gel. Electrophoresis was carried out at 1V/cm for 12h at 4°C. The gel was subjected to in gel hybridization with telomeric probe.

### In gel hybridization

In-gel hybridization was performed as described previously [50, 78]. For native in gel hybridization, gels were hybridized in Denhart’s hybridization buffer with ^32^P-labeled C-/G- telomeric probe. The telomeric probes were prepared as described previously [79]. Gels were washed 3 times with 2×SSC and 0.5% SDS, exposed to PhosphorImager screen (GE Healthcare) and scanned on Typhoon imager (GE Healthcare). Image Quant software was used for data analysis. For denatured in-gel hybridization, gels were denatured with 0.5 M NaOH, neutralized with 1 M Tris–HCl (pH 8.0) and then followed the procedure for native hybridization.

### Western blot

Western blots were performed with antibodies against Flag (Monoclonal ANTI-FLAG M2 antibody, F1804, Sigma), SMARCAL1 (Santa Cruz), RPA2 (EMD Millipore), or β-actin (Proteintech).

### Statistical analysis

Two-tailed unpaired student’s *t*-test was used for statistical analysis (Graphpad Prism). Error bars represent the mean± SEM of three biological repeats/independent experiments. * P<0.05, ** P<0.005, *** P<0.001.

## Supporting information

S1 Fig C-circles and C-overhangs formation is associated with telomere replication. (A) Examination of φ29 DNA polymerase dependent C-circle assay. 100ng U2OS genomic DNA was subjected to C-circle assay in the presence or absence of φ29. Error bars represent the mean ± SEM of three independent experiments. Two-tailed unpaired student’s t-test was used to calculate P-values. **P<0.01.
(B) Standard curve of C-circle assay. 0, 25, 50, 100, 200ng U2OS genomic DNA were input for C-circle assay. Error bars represent the mean ± SEM of three independent experiments. Data were analyzed by linear regression.
(C) C-overhangs are sensitive to RecJf, but resistant to Exo I. U2OS gDNA was digested with RecJf or Exo I, subjected to 2D gel analysis. 5' C-overhangs are indicated by red arrows.
(D) 5' C-overhangs are predominantly present on leading synthesized telomeres. Related to Fig 1H. U2OS cells was pulse-labeled by BrdU for 6hrs after G1/S release. Leading, lagging and unreplicated telomeres were isolated by CsCl gradient ultracentrifugation (data not shown), and subjected to 2D gel analysis. C-overhangs were detected by hybridizing with G-probe under native and denatured condition. 5' C-overhangs are indicated by red arrows.
(PDF)

S2 Fig Replication fork stalling caused by HU or aphidicolin doesn’t lead to enrichment of RPA2 or DNA damage foci at telomeres. (A) HU or aphidicolin treatment (24 h) doesn’t cause increase of RPA2 foci at telomere in U2OS. More than 100 cells were quantified for each experiment. Error bars represent the mean ± SEM of three independent experiments. Two-tailed unpaired student’s t-test was used to calculate P-values. ns: not significant.
(B) HU or aphidicolin treatment (24 h) doesn’t induce TIFs (telomere dysfunction induced foci) in U2OS. 53BP1 was used as an indicator of DNA damage response (DDR). U2OS cells treated with zeocin for 24h were used as a positive control. Telomeric 53BP1 foci were analyzed by IF-FISH. More than 100 cells were analyzed for each experiment. Error bars represent the mean ± SEM of three independent experiments. Two-tailed unpaired student’s t-test was used to calculate P-values. ns: not significant. **P<0.01.
(PDF)

S3 Fig DNA damage induced replication fork collapse during S phase provokes formation of C-circles and 5' C-overhangs. (A) G-overhangs were not altered in U2OS cells treated with HU or aphidicolin (Aphi). Cells were treated for 24hrs, genomic DNA were purified and subjected to 2D gel analysis. G-overhangs are indicated by blue arrows. Values were then normalized with G-overhangs in untreated cells (Ctrl) to obtain relative abundance. Experiments were duplicated and the mean of relative abundance of G-overhangs was indicated.
(B) Zeocin or CPT treatment (24 h) leads to decrease of G-overhangs in U2OS (related to Fig 2D and 2F). Values were then normalized with G-overhangs in untreated cells (Ctrl) to obtain relative abundance. Experiments were duplicated and the mean of relative abundance of G-overhangs was indicated.
(C) Schematic for zeocin treatment of U2OS cells during G1 or mid-S phase. U2OS cells were synchronized at G1/S with double thymidine. Cells were treated with zeocin/DMSO during G1 phase (end of second thymidine block) or during S phase (after 4hrs release from G1/S) for 2hrs.
(D) FACS analysis of U2OS cells treated with DMSO or zeocin during G1 or mid-S phase.
(E) and (F) Zeocin treatment during mid-S phase produces more C-circle and 5' C-overhangs than treatment during G1 phase. Error bars represent the mean ± SEM of three independent experiments.
(G) Zeocin or CPT treatment leads to increase of C-circle in VA13 cells. Error bars represent the mean ± SEM of three independent experiments. Two-tailed unpaired student’s t-test was used to calculate P-values. ***P<0.001.
(H) Zeocin and CPT treatment leads to increase of 5' C-overhangs in VA13 cells. C-overhangs are indicated by red arrows. Values were then normalized with C-overhangs in untreated cells (Ctrl) to obtain relative abundance. Experiments were duplicated and the mean of relative abundance of C-overhangs was indicated.
(PDF)

S4 Fig Replication fork collapse but not fork stalling induces the formation of C-circles and 5' C-overhangs. (A) VP-16 (Topo II poisoner) but not ICRF-187 (Topo II inhibitor) leads to increase of C-overhangs in U2OS cells. Genomic DNA from VP-16 or ICRF-187 treated U2OS cells were digested with restriction enzyme and subjected to 2D gel analysis. G-rich telomeric probe was used to detect C-overhangs. C-overhangs are indicated by red arrows.
(B) VP-16 or ICRF-187 treatment leads to decrease of G-overhangs in U2OS cells. Same as in (A) except that C-rich telomeric probe was used to detect G-overhangs. G-overhangs are indicated by blue arrows.
(C) VP-16 but not ICRF-187 leads to increase of C-circles in U2OS cells. Error bars represent the mean ± SEM of three independent experiments. Two-tailed unpaired student’s t-test was used to calculate P-values. ***P<0.001.
(D)VP-16 but not ICRF-187 treatment (24h) leads to increase of C-overhangs in VA13 cells. Genomic DNA from VP-16 or ICRF-187 treated VA13 cells were digested with restriction enzyme, subjected to 2D gel analysis. G-rich telomeric probe was used to detect C-overhangs. C-overhangs are indicated by red arrows. Values were then normalized with C-overhangs in untreated cells (Ctrl) to obtain relative abundance. Experiments were duplicated and the mean of relative abundance of C-overhangs was indicated.
(E) VP-16 treatment decreases G-overhangs in VA13. Same as in (D) except that C-rich telomeric probe was used to detect G-overhangs. G-overhangs are indicated by blue arrows.
(F) VP-16 but not ICRF-187 leads to increase of C-circles in VA13 cells. Error bars represent the mean ± SEM of three independent experiments. Two-tailed unpaired student’s t-test was used to calculate P-values. ns: not significant. **P<0.01.
(PDF)

S5 Fig CRISPR-Cas9 (sgTel) system inducing ssDNA break in C-rich strand stimulates formation of C-circles and 5' C-overhangs in U2OS. (A) Cells express flag-nuclease-deficient CRISPR-Cas9 (dCas9), wild-type CRISPR-Cas9 (WT) or CRISPR-Cas9 with mutation at RuvC domain (D10A). Western blot of flag showed expression level of indicated Cas9. β-actin was used as a loading control.
(B) Cas9 (sgTel) introduces DNA breaks at telomere. Cells co-expressing indicated Cas9 and sgTel were embedded in agarose plug, lysed and subjected to alkali electrophoresis and hybridization with telomeric C- or G-probe. While intact telomeres stay in plug, DNA fragments released by breaks are able to migrate into the gel and detected by telomeric probe.
(C) Expression of wtCas9 or Cas9-D10A, but not dCas9 results in increase of C-circles. Error bars represent the mean ± SEM of three independent experiments. Two-tailed unpaired student’s t-test was used to calculate P-values. ns: not significant. ***P<0.001.
(D) Expression of wtCas9 or Cas9-D10A, but not dCas9 leads to increase of C-overhangs. C-overhangs are indicated by red arrows.
(PDF)

S6 Fig Deficient replication fork regression promotes C-circles and C-overhangs formation. (A) Knocking down RPA2 (siRPA2) or SMARCAL1 (siSM) in U2OS cells leads to decrease of G-overhangs (related to Fig 5C and 5F). G-overhangs are indicated by blue arrows.
(B) B02 treatment (24hrs) results in decrease of G-overhangs in U2OS (related to Fig 5I).
(C) Western blot shows knockdown efficiency of RPA2 or SMARCAL1 (SM) by siRNA in VA13 cells. β-actin was used as a loading control.
(D) Knockdown of RPA2 (siRPA2) or SMARCAL1 (siSM) leads to increase of C-circles in VA13 cells. Error bars represent the mean ± SEM of three independent experiments. Two-tailed unpaired student’s t-test was used to calculate P-values. **P<0.01, ***P<0.001.
(E) Knockdown of RPA2 (siRPA2) or SMARCAL1 (siSM) leads to increase of C-overhangs in VA13 cells.
(F) Knockdown of RPA2 (siRPA2) or SMARCAL1 (siSM) decreases G-overhangs in VA13 cells.
(G) Inhibition of Rad51 by B02 (24 h) leads to increase of C-circle in VA13 cells. Error bars represent the mean ± SEM of three independent experiments. Two-tailed unpaired student’s t-test was used to calculate P-values. ***P<0.001.
(H) Inhibition of Rad51 by B02 (24 h) leads to increase of C-overhangs in VA13 cells. Values were then normalized with C-overhangs in untreated cells (Ctrl) to obtain relative abundance. Experiments were duplicated and the mean of relative abundance of C-overhangs was indicated.
(I) Inhibition of Rad51 by B02 (24 h) leads to decrease of G-overhangs in VA13 cells. Values were then normalized with G-overhangs in untreated cells (Ctrl) to obtain relative abundance. Experiments were duplicated and the mean of relative abundance of G-overhangs was indicated.
(PDF)

S7 Fig NHEJ machinery is involved in the formation of c-circle. (A) pDNA-PKcs (S2056) was recruited to telomere upon CPT treatment. IF/FISH (Red: Cy3-TelG; Green: pDNA-PKcs (S2056)). U2OS cells were treated with CPT for 24 h, DMSO treated cells were used as a control.
(B) Quantification of (A). Cells with more than two pDNA-PKcs(S2056) foci at telomeres were scored. More than 100 cells were quantified for each experiment. Error bars represent the mean ± SEM of three independent experiments. Two-tailed unpaired student’s t-test was used to calculate P-values.**P<0.01.
(C) NU7441 (pDNA-PKcs (S2056) phosphorylation inhibitor) treatment leads to decrease of C-circles in U2OS cells. VE821 (ATR inhibitor) that is reported to decrease C-circles was used as a control. Error bars represent the mean ± SEM of three independent experiments. Two-tailed unpaired student’s t-test was used to calculate P-values. **P<0.01, ***P<0.001.
(D) and (E) NU7441 has a limited effect on the abundance of C-/G-overhangs in U2OS cells.
(PDF)

S8 Fig Rad51 inhibition leads to telomere replication failure. (A) B02 treatment (24 h) leads to increase of PCNA foci colocalized with telomeres.
(C) B02 treatment (24 h) leads to increase of RPA2 foci colocalized with telomeres.
(B) and (D) Quantification of (A) and (C). More than 100 cells were quantified for each experiment. Error bars represent the mean ± SEM of three independent experiments. Two-tailed unpaired student’s t-test was used to calculate P-values. *P<0.05. ***P<0.001.
(E) B02 treatment suppresses telomere synthesis. G1/S synchronized U2OS cells were released into BrdU containing medium for 9 h in presence or absence (Ctrl) of B02. Genomic DNA was purified and subjected to CsCl gradient ultracentrifugation and slot blot analysis using telomere specific probes.
(F) B02 treatment results in the accumulation of short telomeres in U2OS. U2OS cells were treated with B02 for 0, 2 or 4 days and then subjected to TRF assay.
(PDF)

## References

[1] de Lange T. Shelterin: the protein complex that shapes and safeguards human telomeres. Genes Dev. 2005 9 15;19(18):2100–10. 10.1101/gad.1346005 16166375

[2] Moyzis RK, Buckingham JM, Cram LS, Dani M, Deaven LL, Jones MD, et al A highly conserved repetitive DNA sequence, (TTAGGG)n, present at the telomeres of human chromosomes. Proc Natl Acad Sci U S A. 1988 9;85(18):6622–6. 3413114 10.1073/pnas.85.18.6622 PMC282029

[3] Armanios M, Blackburn EH. The telomere syndromes. Nat Rev Genet. 2012 10;13(10):693–704. 10.1038/nrg3246 22965356 PMC3548426

[4] Watson JD. Origin of concatemeric T7 DNA. Nat New Biol. 1972 10 18;239(94):197–201. 4507727 10.1038/newbio239197a0

[5] Chow TT, Zhao Y, Mak SS, Shay JW, Wright WE. Early and late steps in telomere overhang processing in normal human cells: the position of the final RNA primer drives telomere shortening. Genes Dev. 2012 6 1;26(11):1167–78. 10.1101/gad.187211.112 22661228 PMC3371406

[6] Wu P, Takai H, de Lange T. Telomeric 3' overhangs derive from resection by Exo1 and Apollo and fill-in by POT1b-associated CST. Cell. 2012 7 6;150(1):39–52. 10.1016/j.cell.2012.05.026 22748632 PMC3392515

[7] Smogorzewska A, de Lange T. Regulation of telomerase by telomeric proteins. Annual review of biochemistry. 2004;73:177–208. 10.1146/annurev.biochem.73.071403.160049 15189140

[8] Allsopp RC, Chang E, Kashefi-Aazam M, Rogaev EI, Piatyszek MA, Shay JW, et al Telomere shortening is associated with cell division in vitro and in vivo. Experimental cell research. 1995 9;220(1):194–200. 10.1006/excr.1995.1306 7664836

[9] Shay JW, Wright WE. Senescence and immortalization: role of telomeres and telomerase. Carcinogenesis. 2005 5;26(5):867–74. 10.1093/carcin/bgh296 15471900

[10] Cesare AJ, Reddel RR. Alternative lengthening of telomeres: models, mechanisms and implications. Nat Rev Genet. 2010 5;11(5):319–30. 10.1038/nrg2763 20351727

[11] Pickett HA, Reddel RR. Molecular mechanisms of activity and derepression of alternative lengthening of telomeres. Nat Struct Mol Biol. 2015 11;22(11):875–80. 10.1038/nsmb.3106 26581522

[12] Shay JW, Reddel RR, Wright WE. Cancer. Cancer and telomeres—an ALTernative to telomerase. Science. 2012 6 15;336(6087):1388–90. 10.1126/science.1222394 22700908

[13] Dunham MA, Neumann AA, Fasching CL, Reddel RR. Telomere maintenance by recombination in human cells. Nat Genet. [Research Support, Non-U S Gov't]. 2000;26(4):447–50. 10.1038/82586 11101843

[14] Londono-Vallejo JA, Der-Sarkissian H, Cazes L, Bacchetti S, Reddel RR. Alternative lengthening of telomeres is characterized by high rates of telomeric exchange. Cancer Res. 2004 4 1;64(7):2324–7. 15059879 10.1158/0008-5472.can-03-4035

[15] Yeager TR, Neumann AA, Englezou A, Huschtscha LI, Noble JR, Reddel RR. Telomerase-negative immortalized human cells contain a novel type of promyelocytic leukemia (PML) body. Cancer Res. 1999 9 1;59(17):4175–9. 10485449

[16] Cesare AJ, Griffith JD. Telomeric DNA in ALT cells is characterized by free telomeric circles and heterogeneous t-loops. Mol Cell Biol. 2004 11;24(22):9948–57. 10.1128/MCB.24.22.9948-9957.2004 15509797 PMC525488

[17] Nabetani A, Ishikawa F. Unusual telomeric DNAs in human telomerase-negative immortalized cells. Mol Cell Biol. 2009 2;29(3):703–13. 10.1128/MCB.00603-08 19015236 PMC2630689

[18] Oganesian L, Karlseder J. Mammalian 5' C-rich telomeric overhangs are a mark of recombination-dependent telomere maintenance. Mol Cell. 2011 4 22;42(2):224–36. 10.1016/j.molcel.2011.03.015 21504833 PMC3082866

[19] Cho NW, Dilley RL, Lampson MA, Greenberg RA. Interchromosomal homology searches drive directional ALT telomere movement and synapsis. Cell. 2014 9 25;159(1):108–21. 10.1016/j.cell.2014.08.030 25259924 PMC4177039

[20] Hu Y, Shi G, Zhang L, Li F, Jiang Y, Jiang S, et al Switch telomerase to ALT mechanism by inducing telomeric DNA damages and dysfunction of ATRX and DAXX. Sci Rep. 2016 8 31;6:32280 10.1038/srep32280 27578458 PMC5006076

[21] Poole LA, Zhao R, Glick GG, Lovejoy CA, Eischen CM, Cortez D. SMARCAL1 maintains telomere integrity during DNA replication. Proc Natl Acad Sci U S A. 2015 12 01;112(48):14864–9. 10.1073/pnas.1510750112 26578802 PMC4672769

[22] Huang C, Jia P, Chastain M, Shiva O, Chai W. The human CTC1/STN1/TEN1 complex regulates telomere maintenance in ALT cancer cells. Experimental cell research. 2017 6 15;355(2):95–104. 10.1016/j.yexcr.2017.03.058 28366536 PMC5551977

[23] Flynn RL, Cox KE, Jeitany M, Wakimoto H, Bryll AR, Ganem NJ, et al Alternative lengthening of telomeres renders cancer cells hypersensitive to ATR inhibitors. Science. 2015 1 16;347(6219):273–7. 10.1126/science.1257216 25593184 PMC4358324

[24] Oganesian L, Karlseder J. 5' C-rich telomeric overhangs are an outcome of rapid telomere truncation events. DNA Repair (Amst). 2013 3 01;12(3):238–45. 23347616 10.1016/j.dnarep.2012.12.008 PMC3594334

[25] Lipps HJ, Rhodes D. G-quadruplex structures: in vivo evidence and function. Trends Cell Biol. 2009 8;19(8):414–22. 10.1016/j.tcb.2009.05.002 19589679

[26] Rochette PJ, Brash DE. Human telomeres are hypersensitive to UV-induced DNA Damage and refractory to repair. PLoS Genet. 2010 4 29;6(4):e1000926 10.1371/journal.pgen.1000926 20442874 PMC2861706

[27] Oikawa S, Tada-Oikawa S, Kawanishi S. Site-specific DNA damage at the GGG sequence by UVA involves acceleration of telomere shortening. Biochemistry. 2001 4 17;40(15):4763–8. 11294644 10.1021/bi002721g

[28] Henle ES, Han Z, Tang N, Rai P, Luo Y, Linn S. Sequence-specific DNA cleavage by Fe2+-mediated fenton reactions has possible biological implications. J Biol Chem. 1999 1 08;274(2):962–71. 9873038 10.1074/jbc.274.2.962

[29] Gilson E, Geli V. How telomeres are replicated. Nat Rev Mol Cell Biol. 2007 10;8(10):825–38. 10.1038/nrm2259 17885666

[30] Osborn AJ, Elledge SJ, Zou L. Checking on the fork: the DNA-replication stress-response pathway. Trends Cell Biol. 2002 11;12(11):509–16. 12446112 10.1016/s0962-8924(02)02380-2

[31] Petermann E, Helleday T. Pathways of mammalian replication fork restart. Nat Rev Mol Cell Biol. 2010 10;11(10):683–7. 10.1038/nrm2974 20842177

[32] Yeeles JT, Poli J, Marians KJ, Pasero P. Rescuing stalled or damaged replication forks. Cold Spring Harb Perspect Biol. 2013 5;5(5):a012815 10.1101/cshperspect.a012815 23637285 PMC3632063

[33] Lovejoy CA, Li W, Reisenweber S, Thongthip S, Bruno J, de Lange T, et al Loss of ATRX, genome instability, and an altered DNA damage response are hallmarks of the alternative lengthening of telomeres pathway. PLoS Genet. 2012;8(7):e1002772 10.1371/journal.pgen.1002772 22829774 PMC3400581

[34] Cesare AJ, Kaul Z, Cohen SB, Napier CE, Pickett HA, Neumann AA, et al Spontaneous occurrence of telomeric DNA damage response in the absence of chromosome fusions. Nat Struct Mol Biol. 2009 12;16(12):1244–51. 10.1038/nsmb.1725 19935685

[35] Heaphy CM, de Wilde RF, Jiao Y, Klein AP, Edil BH, Shi C, et al Altered telomeres in tumors with ATRX and DAXX mutations. Science. 2011 7 22;333(6041):425 10.1126/science.1207313 21719641 PMC3174141

[36] Schwartzentruber J, Korshunov A, Liu XY, Jones DT, Pfaff E, Jacob K, et al Driver mutations in histone H3.3 and chromatin remodelling genes in paediatric glioblastoma. Nature. 2012 1 29;482(7384):226–31. 10.1038/nature10833 22286061

[37] Henson JD, Cao Y, Huschtscha LI, Chang AC, Au AY, Pickett HA, et al DNA C-circles are specific and quantifiable markers of alternative-lengthening-of-telomeres activity. Nat Biotechnol. 2009 12;27(12):1181–5. 10.1038/nbt.1587 19935656

[38] Wright WE, Tesmer VM, Liao ML, Shay JW. Normal human telomeres are not late replicating. Experimental cell research. 1999 9 15;251(2):492–9. 10.1006/excr.1999.4602 10471333

[39] Hultdin M, Gronlund E, Norrback KF, Just T, Taneja K, Roos G. Replication timing of human telomeric DNA and other repetitive sequences analyzed by fluorescence in situ hybridization and flow cytometry. Experimental cell research. 2001 12 10;271(2):223–9. 10.1006/excr.2001.5391 11716534

[40] O'Sullivan RJ, Arnoult N, Lackner DH, Oganesian L, Haggblom C, Corpet A, et al Rapid induction of alternative lengthening of telomeres by depletion of the histone chaperone ASF1. Nat Struct Mol Biol. 2014 2;21(2):167–74. 10.1038/nsmb.2754 24413054 PMC3946341

[41] Marechal A, Zou L. RPA-coated single-stranded DNA as a platform for post-translational modifications in the DNA damage response. Cell Res. 2015 1;25(1):9–23. 10.1038/cr.2014.147 25403473 PMC4650586

[42] Zhao Y, Sfeir AJ, Zou Y, Buseman CM, Chow TT, Shay JW, et al Telomere extension occurs at most chromosome ends and is uncoupled from fill-in in human cancer cells. Cell. 2009 8 7;138(3):463–75. 10.1016/j.cell.2009.05.026 19665970 PMC2726829

[43] Min J, Wright WE, Shay JW. Alternative lengthening of telomeres can be maintained by preferential elongation of lagging strands. Nucleic Acids Res. 2017 3 17;45(5):2615–28. 10.1093/nar/gkw1295 28082393 PMC5389697

[44] Baranovskiy AG, Babayeva ND, Suwa Y, Gu J, Pavlov YI, Tahirov TH. Structural basis for inhibition of DNA replication by aphidicolin. Nucleic Acids Res. 2014 12 16;42(22):14013–21. 10.1093/nar/gku1209 25429975 PMC4267640

[45] Petermann E, Orta ML, Issaeva N, Schultz N, Helleday T. Hydroxyurea-stalled replication forks become progressively inactivated and require two different RAD51-mediated pathways for restart and repair. Mol Cell. 2010 2 26;37(4):492–502. 10.1016/j.molcel.2010.01.021 20188668 PMC2958316

[46] Chankova SG, Dimova E, Dimitrova M, Bryant PE. Induction of DNA double-strand breaks by zeocin in Chlamydomonas reinhardtii and the role of increased DNA double-strand breaks rejoining in the formation of an adaptive response. Radiat Environ Biophys. 2007 11;46(4):409–16. 10.1007/s00411-007-0123-2 17639449

[47] Pommier Y. Topoisomerase I inhibitors: camptothecins and beyond. Nat Rev Cancer. 2006 10;6(10):789–802. 10.1038/nrc1977 16990856

[48] Pommier Y. DNA topoisomerase I inhibitors: chemistry, biology, and interfacial inhibition. Chem Rev. 2009 7;109(7):2894–902. 10.1021/cr900097c 19476377 PMC2707511

[49] Nitiss JL. DNA topoisomerase II and its growing repertoire of biological functions. Nat Rev Cancer. 2009 5;9(5):327–37. 10.1038/nrc2608 19377505 PMC2730144

[50] Zhang T, Zhang Z, Li F, Hu Q, Liu H, Tang M, et al Looping-out mechanism for resolution of replicative stress at telomeres. EMBO Rep. 2017 8;18(8):1412–28. 10.15252/embr.201643866 28615293 PMC5538764

[51] Ye J, Lenain C, Bauwens S, Rizzo A, Saint-Leger A, Poulet A, et al TRF2 and apollo cooperate with topoisomerase 2alpha to protect human telomeres from replicative damage. Cell. 2010 7 23;142(2):230–42. 10.1016/j.cell.2010.05.032 20655466

[52] Nitiss JL. Targeting DNA topoisomerase II in cancer chemotherapy. Nat Rev Cancer. 2009 5;9(5):338–50. 10.1038/nrc2607 19377506 PMC2748742

[53] Bailey SM, Cornforth MN, Kurimasa A, Chen DJ, Goodwin EH. Strand-specific postreplicative processing of mammalian telomeres. Science. 2001 9 28;293(5539):2462–5. 10.1126/science.1062560 11577237

[54] Yang Y, Gordenin DA, Resnick MA. A single-strand specific lesion drives MMS-induced hyper-mutability at a double-strand break in yeast. DNA Repair (Amst). 2010 8 5;9(8):914–21. 20663718 10.1016/j.dnarep.2010.06.005 PMC2945237

[55] Roberts SA, Sterling J, Thompson C, Harris S, Mav D, Shah R, et al Clustered mutations in yeast and in human cancers can arise from damaged long single-strand DNA regions. Mol Cell. 2012 5 25;46(4):424–35. 10.1016/j.molcel.2012.03.030 22607975 PMC3361558

[56] Nishimasu H, Ran FA, Hsu PD, Konermann S, Shehata SI, Dohmae N, et al Crystal structure of Cas9 in complex with guide RNA and target DNA. Cell. 2014 2 27;156(5):935–49. 10.1016/j.cell.2014.02.001 24529477 PMC4139937

[57] Jinek M, Jiang F, Taylor DW, Sternberg SH, Kaya E, Ma E, et al Structures of Cas9 endonucleases reveal RNA-mediated conformational activation. Science. 2014 3 14;343(6176):1247997 10.1126/science.1247997 24505130 PMC4184034

[58] Mao P, Liu J, Zhang Z, Zhang H, Liu H, Gao S, et al Homologous recombination-dependent repair of telomeric DSBs in proliferating human cells. Nat Commun. 2016 7 11;7:12154 10.1038/ncomms12154 27396625 PMC4942568

[59] Poole LA, Cortez D. SMARCAL1 and telomeres: Replicating the troublesome ends. Nucleus. 2016 6 29:1–5. 10.1080/19491034.2016.1179413 PMC4991236 27355316

[60] Cox KE, Marechal A, Flynn RL. SMARCAL1 Resolves Replication Stress at ALT Telomeres. Cell Rep. 2016 2 9;14(5):1032–40. 10.1016/j.celrep.2016.01.011 26832416 PMC5051350

[61] Bhat KP, Cortez D. RPA and RAD51: fork reversal, fork protection, and genome stability. Nat Struct Mol Biol. 2018 6;25(6):446–53. 10.1038/s41594-018-0075-z 29807999 PMC6006513

[62] Huang F, Motlekar NA, Burgwin CM, Napper AD, Diamond SL, Mazin AV. Identification of specific inhibitors of human RAD51 recombinase using high-throughput screening. ACS Chem Biol. 2011 6 17;6(6):628–35. 10.1021/cb100428c 21428443 PMC3117970

[63] Drosopoulos WC, Kosiyatrakul ST, Yan Z, Calderano SG, Schildkraut CL. Human telomeres replicate using chromosome-specific, rather than universal, replication programs. J Cell Biol. 2012 4 16;197(2):253–66. 10.1083/jcb.201112083 22508510 PMC3328383

[64] Groth A, Corpet A, Cook AJ, Roche D, Bartek J, Lukas J, et al Regulation of replication fork progression through histone supply and demand. Science. 2007 12 21;318(5858):1928–31. 10.1126/science.1148992 18096807

[65] Zou L, Elledge SJ. Sensing DNA damage through ATRIP recognition of RPA-ssDNA complexes. Science. 2003 6 6;300(5625):1542–8. 10.1126/science.1083430 12791985

[66] Couch FB, Bansbach CE, Driscoll R, Luzwick JW, Glick GG, Betous R, et al ATR phosphorylates SMARCAL1 to prevent replication fork collapse. Genes Dev. 2013 7 15;27(14):1610–23. 10.1101/gad.214080.113 23873943 PMC3731549

[67] Sarbajna S, West SC. Holliday junction processing enzymes as guardians of genome stability. Trends Biochem Sci. 2014 9;39(9):409–19. 10.1016/j.tibs.2014.07.003 25131815

[68] Garcia-Exposito L, Bournique E, Bergoglio V, Bose A, Barroso-Gonzalez J, Zhang S, et al Proteomic Profiling Reveals a Specific Role for Translesion DNA Polymerase eta in the Alternative Lengthening of Telomeres. Cell Rep. 2016 11 08;17(7):1858–71. 10.1016/j.celrep.2016.10.048 27829156 PMC5406014

[69] Diplas BH, He X, Brosnan-Cashman JA, Liu H, Chen LH, Wang Z, et al The genomic landscape of TERT promoter wildtype-IDH wildtype glioblastoma. Nat Commun. 2018 5 25;9(1):2087 10.1038/s41467-018-04448-6 29802247 PMC5970234

[70] Dilley RL, Verma P, Cho NW, Winters HD, Wondisford AR, Greenberg RA. Break-induced telomere synthesis underlies alternative telomere maintenance. Nature. 2016 11 03;539(7627):54–8. 10.1038/nature20099 27760120 PMC5384111

[71] Sanjana NE, Shalem O, Zhang F. Improved vectors and genome-wide libraries for CRISPR screening. Nat Methods. 2014 8;11(8):783–4. 10.1038/nmeth.3047 25075903 PMC4486245

[72] Chen B, Gilbert LA, Cimini BA, Schnitzbauer J, Zhang W, Li GW, et al Dynamic imaging of genomic loci in living human cells by an optimized CRISPR/Cas system. Cell. 2013 12 19;155(7):1479–91. 10.1016/j.cell.2013.12.001 24360272 PMC3918502

[73] Shalem O, Sanjana NE, Hartenian E, Shi X, Scott DA, Mikkelson T, et al Genome-scale CRISPR-Cas9 knockout screening in human cells. Science. 2014 1 3;343(6166):84–7. 10.1126/science.1247005 24336571 PMC4089965

[74] Brewer BJ, Fangman WL. A replication fork barrier at the 3' end of yeast ribosomal RNA genes. Cell. 1988 11 18;55(4):637–43. 3052854 10.1016/0092-8674(88)90222-x

[75] Friedman KL, Brewer BJ. Analysis of replication intermediates by two-dimensional agarose gel electrophoresis. Methods in enzymology. 1995;262:613–27. 8594382 10.1016/0076-6879(95)62048-6

[76] Bailey SM, Goodwin EH, Cornforth MN. Strand-specific fluorescence in situ hybridization: the CO-FISH family. Cytogenet Genome Res. 2004;107(1–2):14–7. 10.1159/000079565 15305050

[77] Sutherland BM, Bennett PV, Sutherland JC. DNA damage quantitation by alkaline gel electrophoresis. Methods Mol Biol. 2006;314:251–73. 10.1385/1-59259-973-7:251 16673887

[78] Ouellette MM, Liao M, Herbert BS, Johnson M, Holt SE, Liss HS, et al Subsenescent telomere lengths in fibroblasts immortalized by limiting amounts of telomerase. J Biol Chem. 2000 4 7;275(14):10072–6. 10744686 10.1074/jbc.275.14.10072

[79] Zhao Y, Shay JW, Wright WE. Telomere G-overhang length measurement method 1: the DSN method. Methods Mol Biol. 2011;735:47–54. 10.1007/978-1-61779-092-8_5 21461810 PMC3528100

