# Supplementary material for: Strand break-induced replication fork collapse leads to C-circles, C-overhangs and telomeric recombination
Source: PLoS Genet. 2019 Feb 4;15(2):e1007925. doi: 10.1371/journal.pgen.1007925 (PMC6382176; doi:10.1371/journal.pgen.1007925)
Supplement: S1 Fig — (A) Examination of φ29 DNA polymerase dependent C-circle assay. 100ng U2OS genomic DNA was subjected to C-circle assay in the presence or absence of φ29. Error bars represent the mean ± SEM of three independent experiments. Two-tailed unpaired student’s t-test was used to calculate P-values. **P<0.01. (B) Standard curve of C-circle assay. 0, 25, 50, 100, 200ng U2OS genomic DNA were input for C-circle assay. Error bars represent the mean ± SEM of three independent experiments. Data were analyzed by linear regression. (C) C-overhangs are sensitive to RecJf, but resistant to Exo I. U2OS gDNA was digested with RecJf or Exo I, subjected to 2D gel analysis. 5' C-overhangs are indicated by red arrows. (D) 5' C-overhangs are predominantly present on leading synthesized telomeres. Related to Fig 1H. U2OS cells was pulse-labeled by BrdU for 6hrs after G1/S release. Leading, lagging and unreplicated telomeres were isolated by CsCl gradient ultracentrifugation (data not shown), and subjected to 2D gel analysis. C-overhangs were detected by hybridizing with G-probe under native and denatured condition. 5' C-overhangs are indicated by red arrows. (PDF) [file pgen.1007925.s001.pdf]

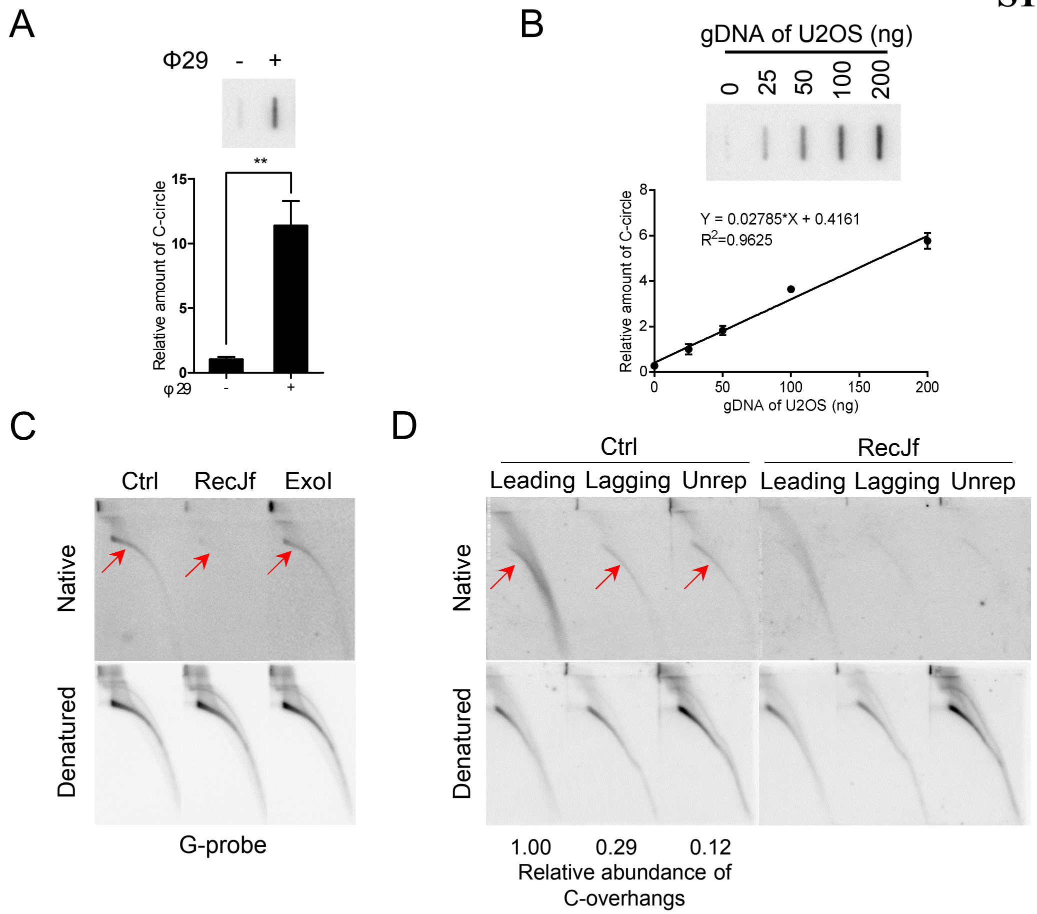

S1 Fig. C-circles and C-overhangs formation is associated with telomere replication.

(A) Examination of φ29 DNA polymerase dependent C-circle assay. 100ng U2OS genomic DNA was subjected to C-circle assay in the presence or absence of φ29. Error bars represent the mean ± SEM of three independent experiments. Two-tailed unpaired student's *t*-test was used to calculate P-values. **P<0.01.

(B) Standard curve of C-circle assay. 0, 25, 50, 100, 200ng U2OS genomic DNA were input for C-circle assay. Error bars represent the mean ± SEM of three independent experiments. Data were analyzed by linear regression.

(C) C-overhangs are sensitive to RecJf, but resistant to Exo I. U2OS gDNA was digested with RecJf or Exo I, subjected to 2D gel analysis. 5' C-overhangs are indicated by red arrows.

(D) 5' C-overhangs are predominantly present on leading synthesized telomeres. Related to Fig 1H. U2OS cells was pulse-labeled by BrdU for 6hrs after G1/S release. Leading, lagging and unreplicated telomeres were isolated by CsCl gradient ultracentrifugation (data not shown), and subjected to 2D gel analysis. C-overhangs were detected by hybridizing with G-probe under native and denatured condition. 5' C-overhangs are indicated by red arrows.
