# Supplementary material for: Strand break-induced replication fork collapse leads to C-circles, C-overhangs and telomeric recombination
Source: PLoS Genet. 2019 Feb 4;15(2):e1007925. doi: 10.1371/journal.pgen.1007925 (PMC6382176; doi:10.1371/journal.pgen.1007925)
Supplement: S2 Fig — (A) HU or aphidicolin treatment (24 h) doesn’t cause increase of RPA2 foci at telomere in U2OS. More than 100 cells were quantified for each experiment. Error bars represent the mean ± SEM of three independent experiments. Two-tailed unpaired student’s t-test was used to calculate P-values. ns: not significant. (B) HU or aphidicolin treatment (24 h) doesn’t induce TIFs (telomere dysfunction induced foci) in U2OS. 53BP1 was used as an indicator of DNA damage response (DDR). U2OS cells treated with zeocin for 24h were used as a positive control. Telomeric 53BP1 foci were analyzed by IF-FISH. More than 100 cells were analyzed for each experiment. Error bars represent the mean ± SEM of three independent experiments. Two-tailed unpaired student’s t-test was used to calculate P-values. ns: not significant. **P<0.01. (PDF) [file pgen.1007925.s002.pdf]

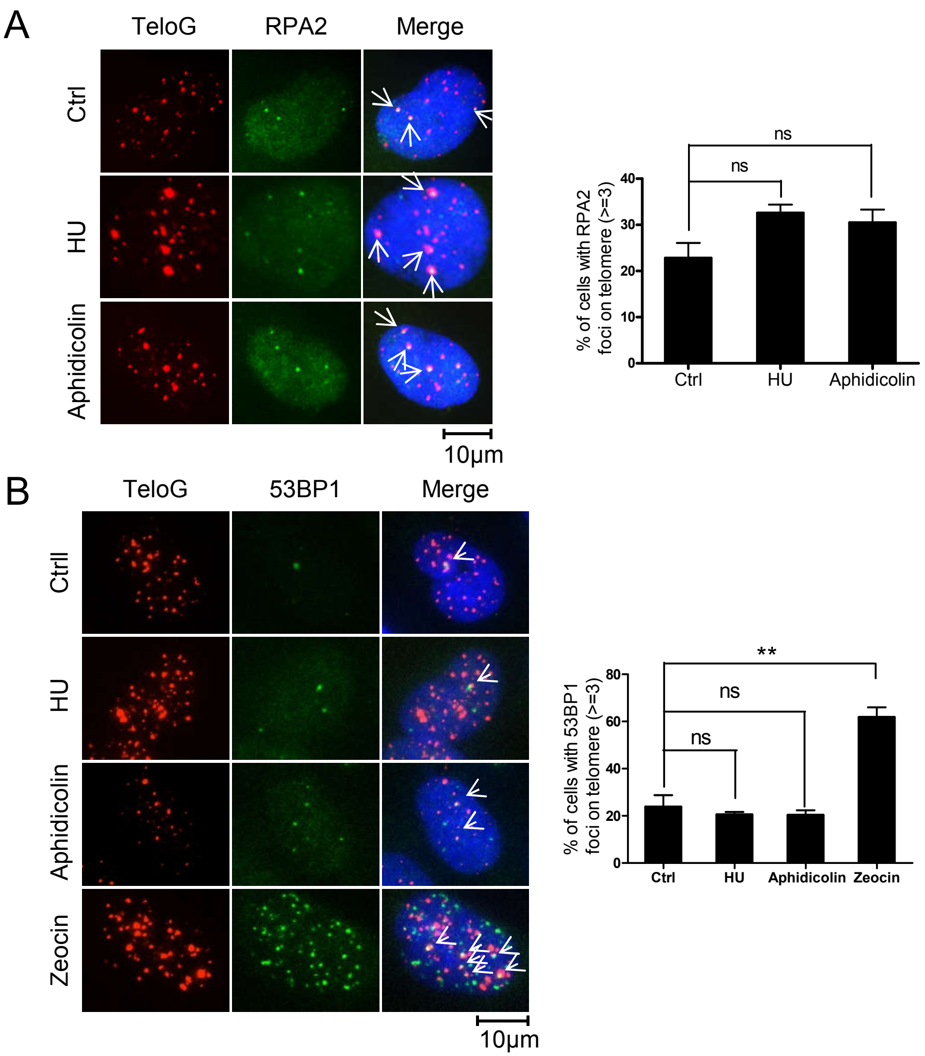

S2 Fig. Replication fork stalling caused by HU or aphidicolin doesn't lead to enrichment of RPA2 or DNA damage foci at telomeres.

(A) HU or aphidicolin treatment (24 h) doesn't cause increase of RPA2 foci at telomere in U2OS. More than 100 cells were quantified for each experiment. Error bars represent the mean ± SEM of three independent experiments. Two-tailed unpaired student's *t*-test was used to calculate P-values. ns: not significant.

(B) HU or aphidicolin treatment (24 h) doesn't induce TIFs (telomere dysfunction induced foci) in U2OS. 53BP1 was used as an indicator of DNA damage response (DDR). U2OS cells treated with zeocin for 24h were used as a positive control. Telomeric 53BP1 foci were analyzed by IF-FISH. More than 100 cells were analyzed for each experiment. Error bars represent the mean ± SEM of three independent experiments. Two-tailed unpaired student's *t*-test was used to calculate P-values. ns: not significant. **$P<0.01$.
