# Supplementary material for: Strand break-induced replication fork collapse leads to C-circles, C-overhangs and telomeric recombination
Source: PLoS Genet. 2019 Feb 4;15(2):e1007925. doi: 10.1371/journal.pgen.1007925 (PMC6382176; doi:10.1371/journal.pgen.1007925)
Supplement: S3 Fig — (A) G-overhangs were not altered in U2OS cells treated with HU or aphidicolin (Aphi). Cells were treated for 24hrs, genomic DNA were purified and subjected to 2D gel analysis. G-overhangs are indicated by blue arrows. Values were then normalized with G-overhangs in untreated cells (Ctrl) to obtain relative abundance. Experiments were duplicated and the mean of relative abundance of G-overhangs was indicated. (B) Zeocin or CPT treatment (24 h) leads to decrease of G-overhangs in U2OS (related to Fig 2D and 2F). Values were then normalized with G-overhangs in untreated cells (Ctrl) to obtain relative abundance. Experiments were duplicated and the mean of relative abundance of G-overhangs was indicated. (C) Schematic for zeocin treatment of U2OS cells during G1 or mid-S phase. U2OS cells were synchronized at G1/S with double thymidine. Cells were treated with zeocin/DMSO during G1 phase (end of second thymidine block) or during S phase (after 4hrs release from G1/S) for 2hrs. (D) FACS analysis of U2OS cells treated with DMSO or zeocin during G1 or mid-S phase. (E) and (F) Zeocin treatment during mid-S phase produces more C-circle and 5' C-overhangs than treatment during G1 phase. Error bars represent the mean ± SEM of three independent experiments. (G) Zeocin or CPT treatment leads to increase of C-circle in VA13 cells. Error bars represent the mean ± SEM of three independent experiments. Two-tailed unpaired student’s t-test was used to calculate P-values. ***P<0.001. (H) Zeocin and CPT treatment leads to increase of 5' C-overhangs in VA13 cells. C-overhangs are indicated by red arrows. Values were then normalized with C-overhangs in untreated cells (Ctrl) to obtain relative abundance. Experiments were duplicated and the mean of relative abundance of C-overhangs was indicated. (PDF) [file pgen.1007925.s003.pdf]

S3 Fig

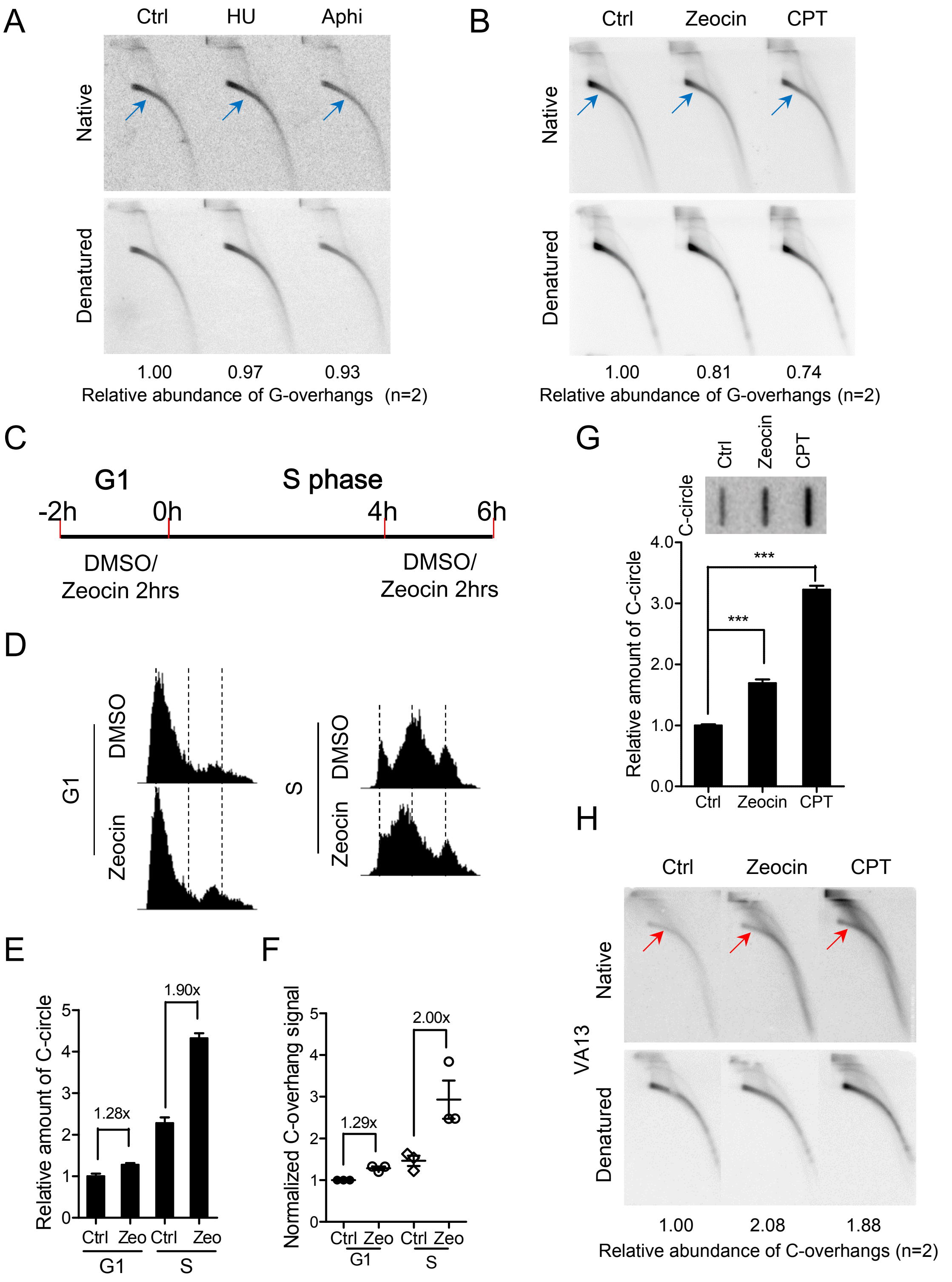

A
Ctrl
HU
Aphi
Native
Denatured
1.00
0.97
0.93
Relative abundance of G-overhangs (n=2)
B
Ctrl
Zeocin
CPT
Native
Denatured
1.00
0.81
0.74
Relative abundance of G-overhangs (n=2)
C
G1
S phase
-2h
0h
4h
6h
DMSO/
Zeocin 2hrs
DMSO/
Zeocin 2hrs
D
G1
DMSO
Zeocin
S
DMSO
Zeocin
E
Relative amount of C-circle
1.28x
1.90x
Ctrl
Zeo
Ctrl
Zeo
G1
S
F
Normalized C-overhang signal
1.29x
2.00x
Ctrl
Zeo
Ctrl
Zeo
G1
S
G
Ctrl
Zeocin
CPT
C-circle
Relative amount of C-circle
***
***
Ctrl
Zeocin
CPT
H
Ctrl
Zeocin
CPT
VA13
Native
Denatured
1.00
2.08
1.88
Relative abundance of C-overhangs (n=2)

S3 Fig. DNA damage induced replication fork collapse during S phase provokes formation of C-circles and 5' C-overhangs.

(A) G-overhangs were not altered in U2OS cells treated with HU or aphidicolin (Aphi). Cells were treated for 24hrs, genomic DNA were purified and subjected to 2D gel analysis. G-overhangs are indicated by blue arrows. Values were then normalized with G-overhangs in untreated cells (Ctrl) to obtain relative abundance. Experiments were duplicated and the mean of relative abundance of G-overhangs was indicated.

(B) Zeocin or CPT treatment (24 h) leads to decrease of G-overhangs in U2OS (related to **Fig 2D** and **2F**). Values were then normalized with G-overhangs in untreated cells (Ctrl) to obtain relative abundance. Experiments were duplicated and the mean of relative abundance of G-overhangs was indicated.

(C) Schematic for zeocin treatment of U2OS cells during G1 or mid-S phase. U2OS cells were synchronized at G1/S with double thymidine. Cells were treated with zeocin/DMSO during G1 phase (end of second thymidine block) or during S phase (after 4hrs release from G1/S) for 2hrs.

(D) FACS analysis of U2OS cells treated with DMSO or zeocin during G1 or mid-S phase.

(E) and **(F)** Zeocin treatment during mid-S phase produces more C-circle and 5' C-overhangs than treatment during G1 phase. Error bars represent the mean ± SEM of three independent experiments.

(G) Zeocin or CPT treatment leads to increase of C-circle in VA13 cells. Error bars represent the mean ± SEM of three independent experiments. Two-tailed unpaired student's *t*-test was used to calculate P-values. ***P<0.001.

(H) Zeocin and CPT treatment leads to increase of 5' C-overhangs in VA13 cells. C-overhangs are indicated by red arrows. Values were then normalized with C-overhangs in untreated cells (Ctrl) to obtain relative abundance. Experiments were duplicated and the mean of relative abundance of C-overhangs was indicated.
