# Supplementary material for: Strand break-induced replication fork collapse leads to C-circles, C-overhangs and telomeric recombination
Source: PLoS Genet. 2019 Feb 4;15(2):e1007925. doi: 10.1371/journal.pgen.1007925 (PMC6382176; doi:10.1371/journal.pgen.1007925)
Supplement: S4 Fig — (A) VP-16 (Topo II poisoner) but not ICRF-187 (Topo II inhibitor) leads to increase of C-overhangs in U2OS cells. Genomic DNA from VP-16 or ICRF-187 treated U2OS cells were digested with restriction enzyme and subjected to 2D gel analysis. G-rich telomeric probe was used to detect C-overhangs. C-overhangs are indicated by red arrows. (B) VP-16 or ICRF-187 treatment leads to decrease of G-overhangs in U2OS cells. Same as in (A) except that C-rich telomeric probe was used to detect G-overhangs. G-overhangs are indicated by blue arrows. (C) VP-16 but not ICRF-187 leads to increase of C-circles in U2OS cells. Error bars represent the mean ± SEM of three independent experiments. Two-tailed unpaired student’s t-test was used to calculate P-values. ***P<0.001. (D)VP-16 but not ICRF-187 treatment (24h) leads to increase of C-overhangs in VA13 cells. Genomic DNA from VP-16 or ICRF-187 treated VA13 cells were digested with restriction enzyme, subjected to 2D gel analysis. G-rich telomeric probe was used to detect C-overhangs. C-overhangs are indicated by red arrows. Values were then normalized with C-overhangs in untreated cells (Ctrl) to obtain relative abundance. Experiments were duplicated and the mean of relative abundance of C-overhangs was indicated. (E) VP-16 treatment decreases G-overhangs in VA13. Same as in (D) except that C-rich telomeric probe was used to detect G-overhangs. G-overhangs are indicated by blue arrows. (F) VP-16 but not ICRF-187 leads to increase of C-circles in VA13 cells. Error bars represent the mean ± SEM of three independent experiments. Two-tailed unpaired student’s t-test was used to calculate P-values. ns: not significant. **P<0.01. (PDF) [file pgen.1007925.s004.pdf]

S4 Fig

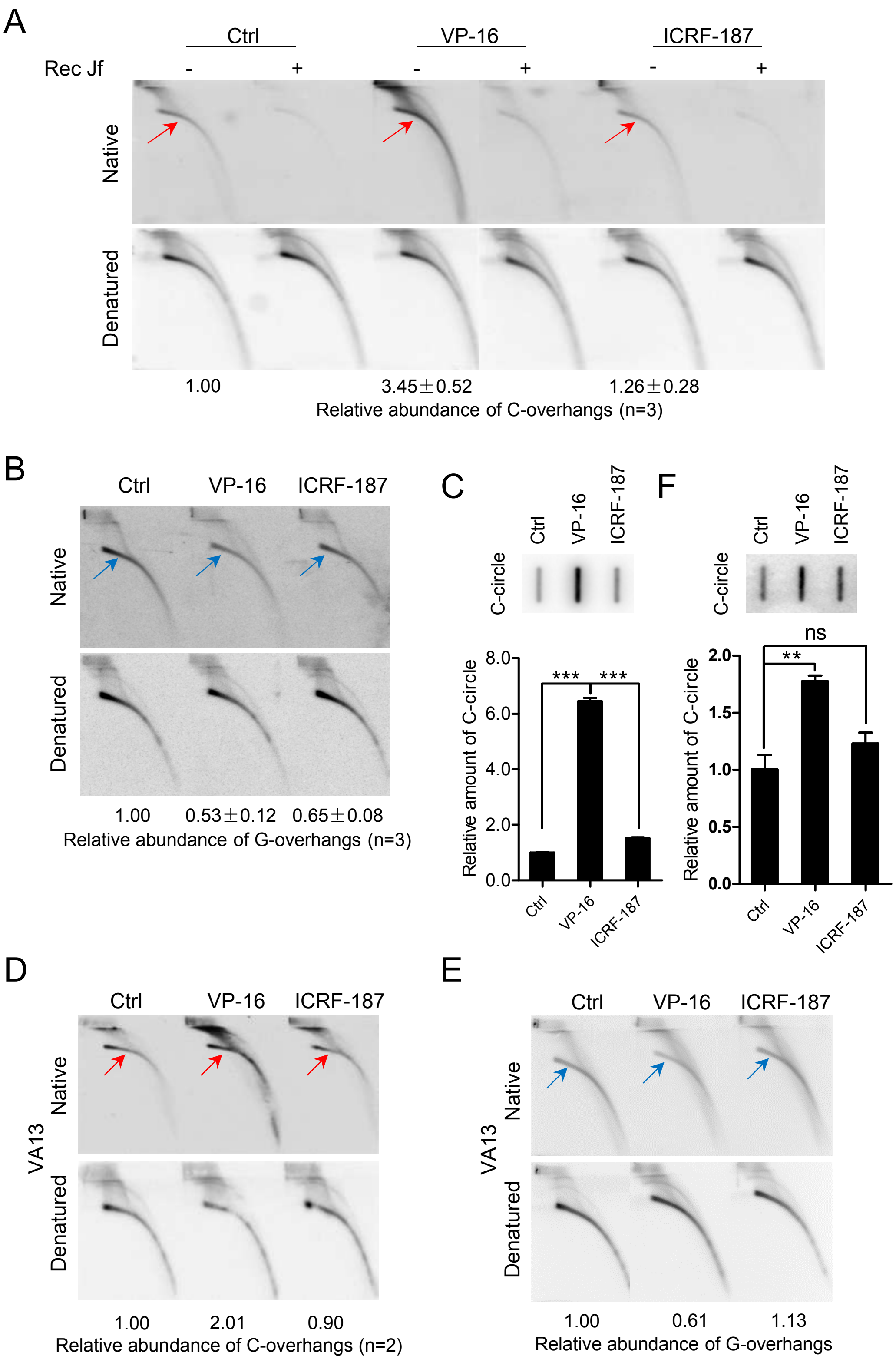

A
Ctrl
VP-16
ICRF-187
Rec Jf
-
+
Native
Denatured
1.00
3.45±0.52
1.26±0.28
Relative abundance of C-overhangs (n=3)
B
Ctrl
VP-16
ICRF-187
Native
Denatured
1.00
0.53±0.12
0.65±0.08
Relative abundance of G-overhangs (n=3)
C
Ctrl
VP-16
ICRF-187
C-circle
Relative amount of C-circle
8.0
6.0
4.0
2.0
0.0
***
***
F
C-circle
Relative amount of C-circle
2.0
1.5
1.0
0.5
0.0
ns
**
D
VA13
Native
Denatured
1.00
2.01
0.90
Relative abundance of C-overhangs (n=2)
E
VA13
1.00
0.61
1.13
Relative abundance of G-overhangs

S4 Fig. Replication fork collapse but not fork stalling induces the formation of C-circles and 5' C-overhangs.

(A) VP-16 (Topo II poisoner) but not ICRF-187 (Topo II inhibitor) leads to increase of C-overhangs in U2OS cells. Genomic DNA from VP-16 or ICRF-187 treated U2OS cells were digested with restriction enzyme and subjected to 2D gel analysis. G-rich telomeric probe was used to detect C-overhangs. C-overhangs are indicated by red arrows.

(B) VP-16 or ICRF-187 treatment leads to decrease of G-overhangs in U2OS cells. Same as in (A) except that C-rich telomeric probe was used to detect G-overhangs. G-overhangs are indicated by blue arrows.

(C) VP-16 but not ICRF-187 leads to increase of C-circles in U2OS cells. Error bars represent the mean ± SEM of three independent experiments. Two-tailed unpaired student's *t*-test was used to calculate P-values. ***P<0.001

(D)VP-16 but not ICRF-187 treatment (24h) leads to increase of C-overhangs in VA13 cells. Genomic DNA from VP-16 or ICRF-187 treated VA13 cells were digested with restriction enzyme, subjected to 2D gel analysis. G-rich telomeric probe was used to detect C-overhangs. C-overhangs are indicated by red arrows. Values were then normalized with C-overhangs in untreated cells (Ctrl) to obtain relative abundance. Experiments were duplicated and the mean of relative abundance of C-overhangs was indicated.

(E) VP-16 treatment decreases G-overhangs in VA13. Same as in (**D**) except that C-rich telomeric probe was used to detect G-overhangs. G-overhangs are indicated by blue arrows.

(F) VP-16 but not ICRF-187 leads to increase of C-circles in VA13 cells. Error bars represent the mean ± SEM of three independent experiments. Two-tailed unpaired student's *t*-test was used to calculate P-values. ns: not significant. **P<0.01.
