# Supplementary material for: Strand break-induced replication fork collapse leads to C-circles, C-overhangs and telomeric recombination
Source: PLoS Genet. 2019 Feb 4;15(2):e1007925. doi: 10.1371/journal.pgen.1007925 (PMC6382176; doi:10.1371/journal.pgen.1007925)
Supplement: S5 Fig — (A) Cells express flag-nuclease-deficient CRISPR-Cas9 (dCas9), wild-type CRISPR-Cas9 (WT) or CRISPR-Cas9 with mutation at RuvC domain (D10A). Western blot of flag showed expression level of indicated Cas9. β-actin was used as a loading control. (B) Cas9 (sgTel) introduces DNA breaks at telomere. Cells co-expressing indicated Cas9 and sgTel were embedded in agarose plug, lysed and subjected to alkali electrophoresis and hybridization with telomeric C- or G-probe. While intact telomeres stay in plug, DNA fragments released by breaks are able to migrate into the gel and detected by telomeric probe. (C) Expression of wtCas9 or Cas9-D10A, but not dCas9 results in increase of C-circles. Error bars represent the mean ± SEM of three independent experiments. Two-tailed unpaired student’s t-test was used to calculate P-values. ns: not significant. ***P<0.001. (D) Expression of wtCas9 or Cas9-D10A, but not dCas9 leads to increase of C-overhangs. C-overhangs are indicated by red arrows. (PDF) [file pgen.1007925.s005.pdf]

S5 Fig

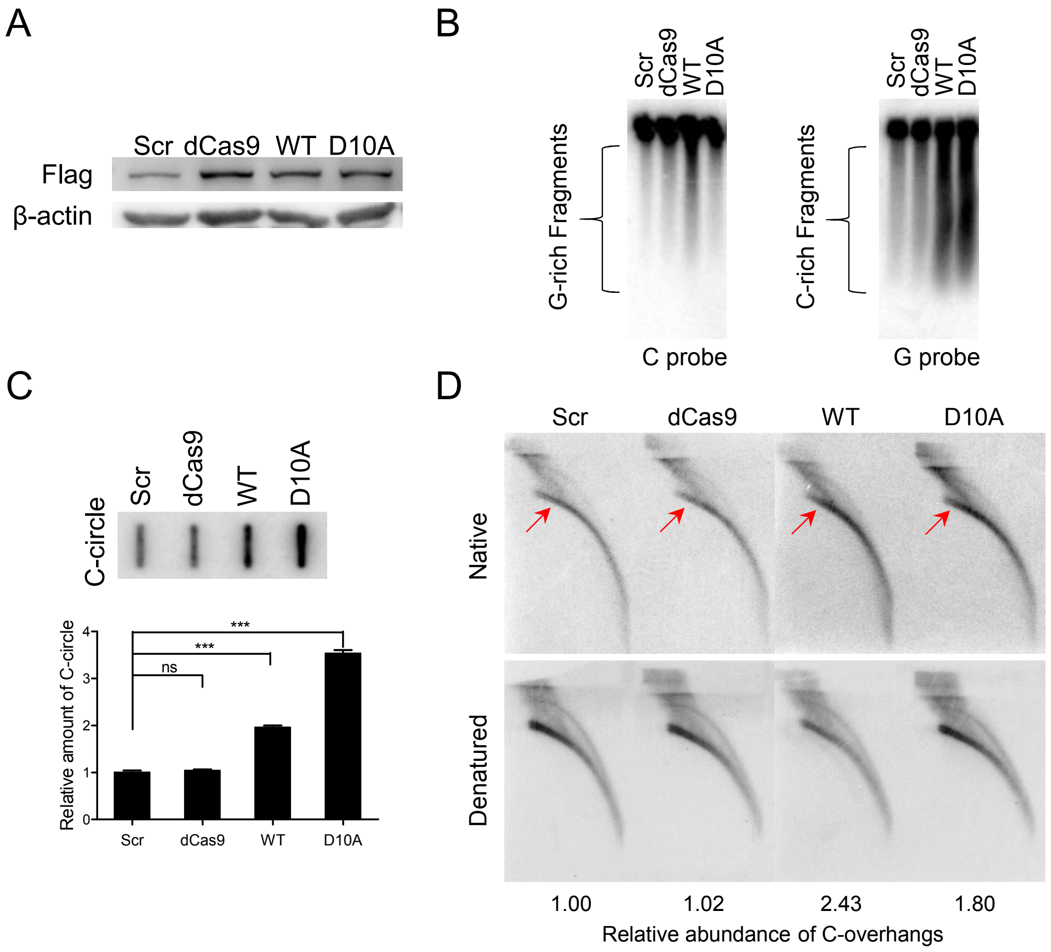

S5 Fig. CRISPR-Cas9 (sgTel) system inducing ssDNA break in C-rich strand stimulates formation of C-circles and 5' C-overhangs in U2OS.

(A) Cells express flag-nuclease-deficient CRISPR-Cas9 (dCas9), wild-type CRISPR-Cas9 (WT) or CRISPR-Cas9 with mutation at RuvC domain (D10A). Western blot of flag showed expression level of indicated Cas9. β-actin was used as a loading control.

(B) Cas9 (sgTel) introduces DNA breaks at telomere. Cells co-expressing indicated Cas9 and sgTel were embedded in agarose plug, lysed and subjected to alkali electrophoresis and hybridization with telomeric C- or G-probe. While intact telomeres stay in plug, DNA fragments released by breaks are able to migrate into the gel and detected by telomeric probe.

(C) Expression of wtCas9 or Cas9-D10A, but not dCas9 results in increase of C-circles. Error bars represent the mean ± SEM of three independent experiments. Two-tailed unpaired student's *t*-test was used to calculate P-values. ns: not significant. ***P<0.001.

(D) Expression of wtCas9 or Cas9-D10A, but not dCas9 leads to increase of C-overhangs. C-overhangs are indicated by red arrows.
