# Supplementary material for: Strand break-induced replication fork collapse leads to C-circles, C-overhangs and telomeric recombination
Source: PLoS Genet. 2019 Feb 4;15(2):e1007925. doi: 10.1371/journal.pgen.1007925 (PMC6382176; doi:10.1371/journal.pgen.1007925)
Supplement: S6 Fig — (A) Knocking down RPA2 (siRPA2) or SMARCAL1 (siSM) in U2OS cells leads to decrease of G-overhangs (related to Fig 5C and 5F). G-overhangs are indicated by blue arrows. (B) B02 treatment (24hrs) results in decrease of G-overhangs in U2OS (related to Fig 5I). (C) Western blot shows knockdown efficiency of RPA2 or SMARCAL1 (SM) by siRNA in VA13 cells. β-actin was used as a loading control. (D) Knockdown of RPA2 (siRPA2) or SMARCAL1 (siSM) leads to increase of C-circles in VA13 cells. Error bars represent the mean ± SEM of three independent experiments. Two-tailed unpaired student’s t-test was used to calculate P-values. **P<0.01, ***P<0.001. (E) Knockdown of RPA2 (siRPA2) or SMARCAL1 (siSM) leads to increase of C-overhangs in VA13 cells. (F) Knockdown of RPA2 (siRPA2) or SMARCAL1 (siSM) decreases G-overhangs in VA13 cells. (G) Inhibition of Rad51 by B02 (24 h) leads to increase of C-circle in VA13 cells. Error bars represent the mean ± SEM of three independent experiments. Two-tailed unpaired student’s t-test was used to calculate P-values. ***P<0.001. (H) Inhibition of Rad51 by B02 (24 h) leads to increase of C-overhangs in VA13 cells. Values were then normalized with C-overhangs in untreated cells (Ctrl) to obtain relative abundance. Experiments were duplicated and the mean of relative abundance of C-overhangs was indicated. (I) Inhibition of Rad51 by B02 (24 h) leads to decrease of G-overhangs in VA13 cells. Values were then normalized with G-overhangs in untreated cells (Ctrl) to obtain relative abundance. Experiments were duplicated and the mean of relative abundance of G-overhangs was indicated. (PDF) [file pgen.1007925.s006.pdf]

S6 Fig

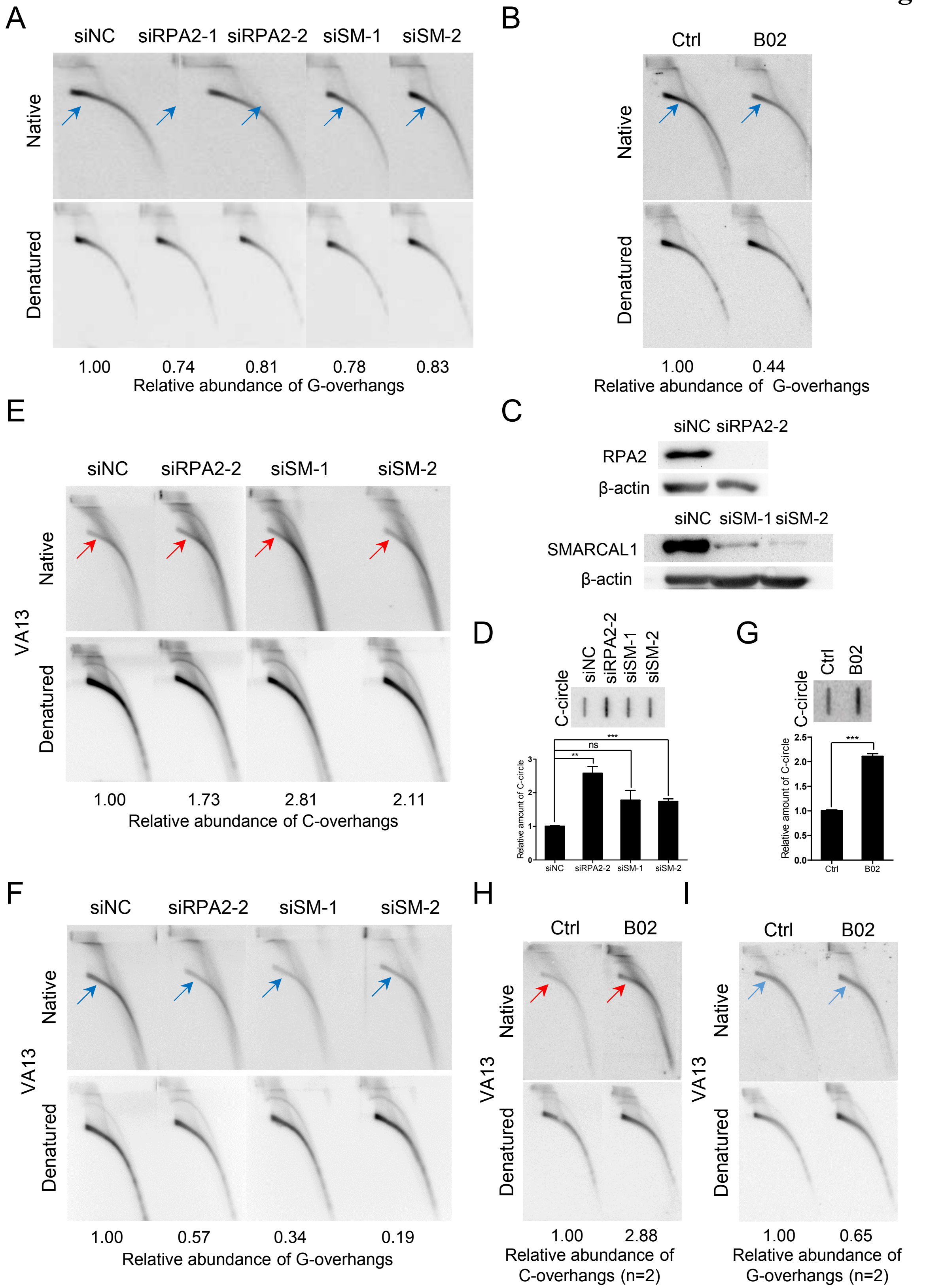
A
siNC
siRPA2-1
siRPA2-2
siSM-1
siSM-2
Native
Denatured
1.00
0.74
0.81
0.78
0.83
Relative abundance of G-overhangs
B
Ctrl
B02
Native
Denatured
1.00
0.44
Relative abundance of G-overhangs
C
siNC siRPA2-2
RPA2
β-actin
siNC siSM-1 siSM-2
SMARCAL1
β-actin
D
C-circle
siNC
siRPA2-2
siSM-1
siSM-2
Relative amount of C-circle
ns
**
***
E
siNC
siRPA2-2
siSM-1
siSM-2
VA13
Native
Denatured
1.00
1.73
2.81
2.11
Relative abundance of C-overhangs
F
siNC
siRPA2-2
siSM-1
siSM-2
VA13
Native
Denatured
1.00
0.57
0.34
0.19
Relative abundance of G-overhangs
G
C-circle
Ctrl
B02
Relative amount of C-circle
***
H
Ctrl
B02
VA13
Native
Denatured
1.00
2.88
Relative abundance of C-overhangs (n=2)
I
Ctrl
B02
VA13
Native
Denatured
1.00
0.65
Relative abundance of G-overhangs (n=2)

S6 Fig. Deficient replication fork regression promotes C-circles and C-overhangs formation.

(A) Knocking down RPA2 (siRPA2) or SMARCAL1 (siSM) in U2OS cells leads to decrease of G-overhangs (related to **Fig 5C** and **5F**). G-overhangs are indicated by blue arrows.

(B) B02 treatment (24hrs) results in decrease of G-overhangs in U2OS (related to **Fig 5I**).

(C) Western blot shows knockdown efficiency of RPA2 or SMARCAL1 (SM) by siRNA in VA13 cells. β-actin was used as a loading control.

(D) Knockdown of RPA2 (siRPA2) or SMARCAL1 (siSM) leads to increase of C-circles in VA13 cells. Error bars represent the mean ± SEM of three independent experiments. Two-tailed unpaired student's *t*-test was used to calculate P-values. **P<0.01, ***P<0.001.

(E) Knockdown of RPA2 (siRPA2) or SMARCAL1 (siSM) leads to increase of C-overhangs in VA13 cells.

(F) Knockdown of RPA2 (siRPA2) or SMARCAL1 (siSM) decreases G-overhangs in VA13 cells.

(G) Inhibition of Rad51 by B02 (24 h) leads to increase of C-circle in VA13 cells. Error bars represent the mean ± SEM of three independent experiments. Two-tailed unpaired student's *t*-test was used to calculate P-values. ***P<0.001.

(H) Inhibition of Rad51 by B02 (24 h) leads to increase of C-overhangs in VA13 cells. Values were then normalized with C-overhangs in untreated cells (Ctrl) to obtain relative abundance. Experiments were duplicated and the mean of relative abundance of C-overhangs was indicated.

(I) Inhibition of Rad51 by B02 (24 h) leads to decrease of G-overhangs in VA13 cells. Values were then normalized with G-overhangs in untreated cells (Ctrl) to obtain relative abundance. Experiments were duplicated and the mean of relative abundance of G-overhangs was indicated.
