# Supplementary material for: Strand break-induced replication fork collapse leads to C-circles, C-overhangs and telomeric recombination
Source: PLoS Genet. 2019 Feb 4;15(2):e1007925. doi: 10.1371/journal.pgen.1007925 (PMC6382176; doi:10.1371/journal.pgen.1007925)
Supplement: S7 Fig — (A) pDNA-PKcs (S2056) was recruited to telomere upon CPT treatment. IF/FISH (Red: Cy3-TelG; Green: pDNA-PKcs (S2056)). U2OS cells were treated with CPT for 24 h, DMSO treated cells were used as a control. (B) Quantification of (A). Cells with more than two pDNA-PKcs(S2056) foci at telomeres were scored. More than 100 cells were quantified for each experiment. Error bars represent the mean ± SEM of three independent experiments. Two-tailed unpaired student’s t-test was used to calculate P-values.**P<0.01. (C) NU7441 (pDNA-PKcs (S2056) phosphorylation inhibitor) treatment leads to decrease of C-circles in U2OS cells. VE821 (ATR inhibitor) that is reported to decrease C-circles was used as a control. Error bars represent the mean ± SEM of three independent experiments. Two-tailed unpaired student’s t-test was used to calculate P-values. **P<0.01, ***P<0.001. (D) and (E) NU7441 has a limited effect on the abundance of C-/G-overhangs in U2OS cells. (PDF) [file pgen.1007925.s007.pdf]

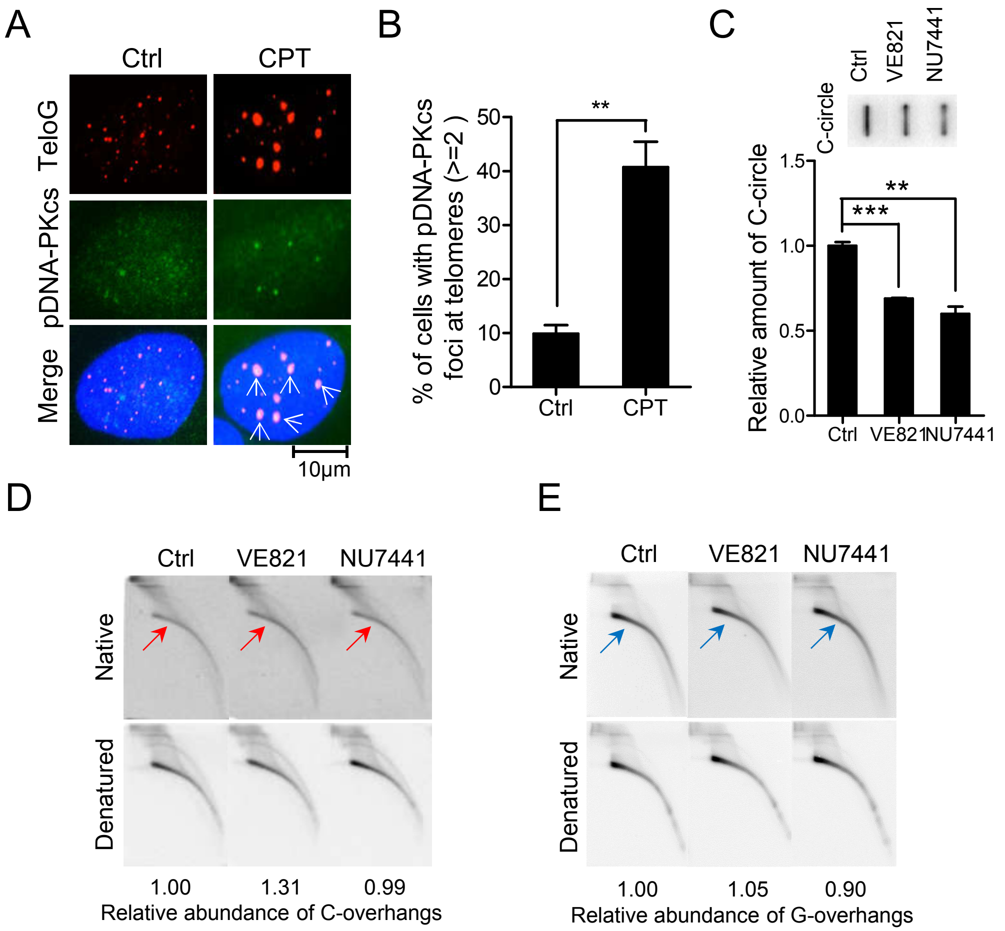

S7 Fig. NHEJ machinery is involved in the formation of c-circle.

(A) pDNA-PKcs (S2056) was recruited to telomere upon CPT treatment. IF/FISH (Red: Cy3-TelG; Green: pDNA-PKcs (S2056)). U2OS cells were treated with CPT for 24 h, DMSO treated cells were used as a control.

(B) Quantification of (A). Cells with more than two pDNA-PKcs(S2056) foci at telomeres were scored. More than 100 cells were quantified for each experiment. Error bars represent the mean ± SEM of three independent experiments. Two-tailed unpaired student's *t*-test was used to calculate P-values.**P<0.01.

(C) NU7441 (pDNA-PKcs (S2056) phosphorylation inhibitor) treatment leads to decrease of C-circles in U2OS cells. VE821 (ATR inhibitor) that is reported to decrease C-circles was used as a control. Error bars represent the mean ± SEM of three independent experiments. Two-tailed unpaired student's *t*-test was used to calculate P-values. **P<0.01, ***P<0.001.

(D) and **(E)** NU7441 has a limited effect on the abundance of C-/G-overhangs in U2OS cells.
