# Supplementary material for: Strand break-induced replication fork collapse leads to C-circles, C-overhangs and telomeric recombination
Source: PLoS Genet. 2019 Feb 4;15(2):e1007925. doi: 10.1371/journal.pgen.1007925 (PMC6382176; doi:10.1371/journal.pgen.1007925)
Supplement: S8 Fig — (A) B02 treatment (24 h) leads to increase of PCNA foci colocalized with telomeres. (C) B02 treatment (24 h) leads to increase of RPA2 foci colocalized with telomeres. (B) and (D) Quantification of (A) and (C). More than 100 cells were quantified for each experiment. Error bars represent the mean ± SEM of three independent experiments. Two-tailed unpaired student’s t-test was used to calculate P-values. *P<0.05. ***P<0.001. (E) B02 treatment suppresses telomere synthesis. G1/S synchronized U2OS cells were released into BrdU containing medium for 9 h in presence or absence (Ctrl) of B02. Genomic DNA was purified and subjected to CsCl gradient ultracentrifugation and slot blot analysis using telomere specific probes. (F) B02 treatment results in the accumulation of short telomeres in U2OS. U2OS cells were treated with B02 for 0, 2 or 4 days and then subjected to TRF assay. (PDF) [file pgen.1007925.s008.pdf]

S8 Fig

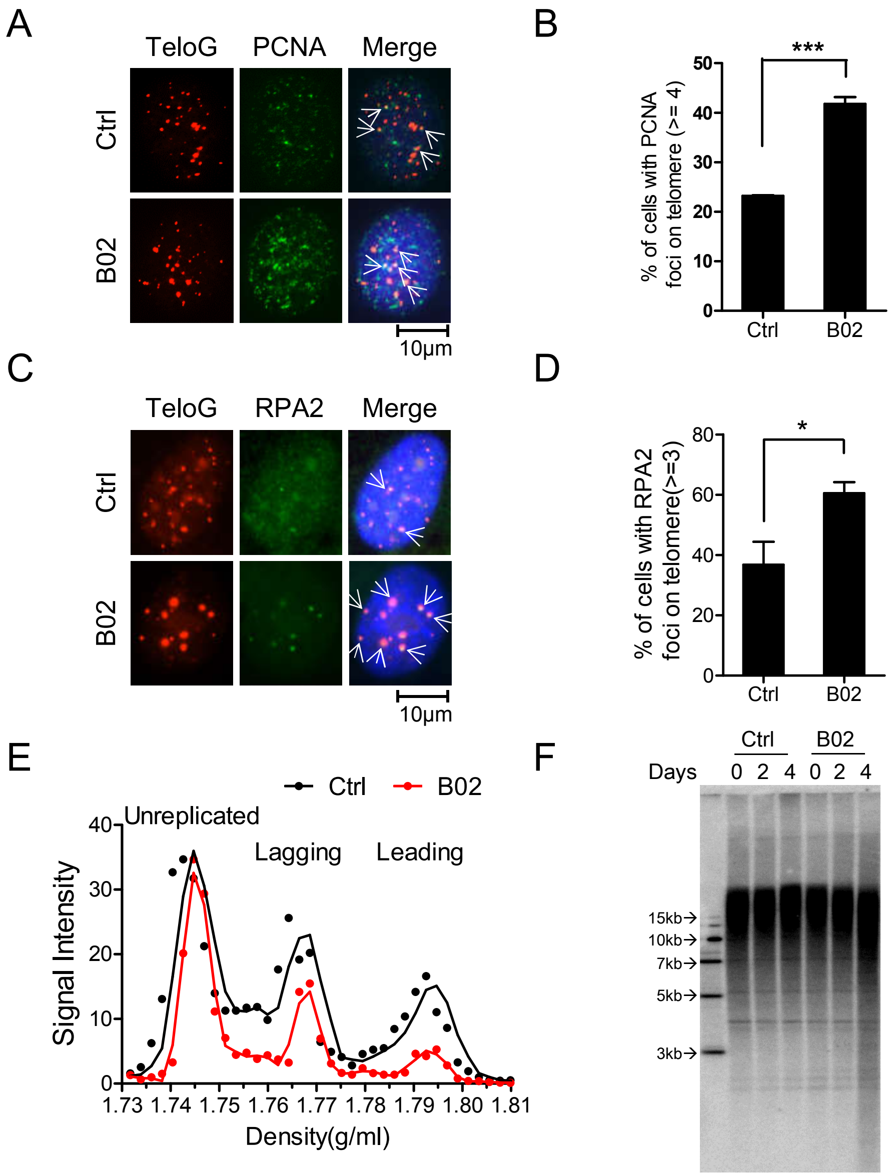

S8 Fig. Rad51 inhibition leads to telomere replication failure.

(A) B02 treatment (24 h) leads to increase of PCNA foci colocalized with telomeres.

(C) B02 treatment (24 h) leads to increase of RPA2 foci colocalized with telomeres.

(B) and **(D)** Quantification of (A) and (C). More than 100 cells were quantified for each experiment. Error bars represent the mean ± SEM of three independent experiments. Two-tailed unpaired student's *t*-test was used to calculate P-values. *P<0.05. ***P<0.001.

(E) B02 treatment suppresses telomere synthesis. G1/S synchronized U2OS cells were released into BrdU containing medium for 9 h in presence or absence (Ctrl) of B02. Genomic DNA was purified and subjected to CsCl gradient ultracentrifugation and slot blot analysis using telomere specific probes.

(F) B02 treatment results in the accumulation of short telomeres in U2OS. U2OS cells were treated with B02 for 0, 2 or 4 days and then subjected to TRF assay.
